# Histidine supplementation can escalate or rescue HARS deficiency in a Charcot–Marie–Tooth disease model

**DOI:** 10.1093/hmg/ddac239

**Published:** 2022-09-26

**Authors:** Yi Qiu, Rosan Kenana, Aruun Beharry, Sarah D P Wilhelm, Sung Yuan Hsu, Victoria M Siu, Martin Duennwald, Ilka U Heinemann

**Affiliations:** Department of Biochemistry, The University of Western Ontario, London, ON N6A 5C1, Canada; Department of Biochemistry, The University of Western Ontario, London, ON N6A 5C1, Canada; Department of Biochemistry, The University of Western Ontario, London, ON N6A 5C1, Canada; Department of Biochemistry, The University of Western Ontario, London, ON N6A 5C1, Canada; Department of Biochemistry, The University of Western Ontario, London, ON N6A 5C1, Canada; Department of Biochemistry, The University of Western Ontario, London, ON N6A 5C1, Canada; Department of Anatomy and Cell Biology, The University of Western Ontario, London, ON N6A 5C1, Canada; Department of Biochemistry, The University of Western Ontario, London, ON N6A 5C1, Canada

## Abstract

Aminoacyl-tRNA synthetases are essential enzymes responsible for charging amino acids onto cognate tRNAs during protein synthesis. In histidyl-tRNA synthetase (HARS), autosomal dominant mutations V133F, V155G, Y330C and S356N in the HARS catalytic domain cause Charcot–Marie–Tooth disease type 2 W (CMT2W), while tRNA-binding domain mutation Y454S causes recessive Usher syndrome type IIIB. In a yeast model, all human HARS variants complemented a genomic deletion of the yeast ortholog HTS1 at high expression levels. CMT2W associated mutations, but not Y454S, resulted in reduced growth. We show mistranslation of histidine to glutamine and threonine in V155G and S356N but not Y330C mutants in yeast. Mistranslating V155G and S356N mutants lead to accumulation of insoluble proteins, which was rescued by histidine. Mutants V133F and Y330C showed the most significant growth defect and decreased HARS abundance in cells. Here, histidine supplementation led to insoluble protein aggregation and further reduced viability, indicating histidine toxicity associated with these mutants. V133F proteins displayed reduced thermal stability *in vitro,* which was rescued by tRNA. Our data will inform future treatment options for HARS patients, where histidine supplementation may either have a toxic or compensating effect depending on the nature of the causative HARS variant.

## Introduction

Charcot–Marie–Tooth (CMT) disease is a heterogeneous genetic disorder that leads to progressive chronic neuropathy affecting the motor and sensory nerves. CMT is the most common inherited neurological disease with a prevalence of 1 in 2500 people. The onset of disease phenotype occurs in the first or second decade of life (1), and treatment is restricted to alleviating CMT symptoms (2).

Aminoacyl-tRNA synthetases (ARSs) are essential enzymes responsible for synthesizing properly aminoacylated tRNAs that are the key substrates the ribosome uses to translate the genetic code (3). Several ARSs have been associated with CMT peripheral neuropathy, namely YARS (4), AARS (5), GARS (6), HARS (7) and WARS (8), and potentially MARS (9). HARS is specifically associated with Charcot–Marie–Tooth disease type 2 W (CMT2W), an autosomal dominant axonal-form subtype of CMT. Most patients present with gait difficulties, foot deformities and distal sensory impairments (7). Experimental evidence has shown that both loss-of-function and gain-of-function of different ARS variants can lead to CMT phenotypes, and both mechanisms may not be mutually exclusive. HARS-linked CMT disease may be caused by either reduced aminoacylation of tRNA^His^ (10) or a relaxed conformation-driven mechanism (11). Furthermore, in zebrafish, knockdown of HARS leads to cell cycle arrest and apoptosis of neuronal progenitor cells (12). Further investigation into CMT-ARS pathogenesis is required to determine the mechanisms by which HARS variants cause disease, which is essential for the development of effective treatments for this irreversible class of illnesses.

**Figure 1:**
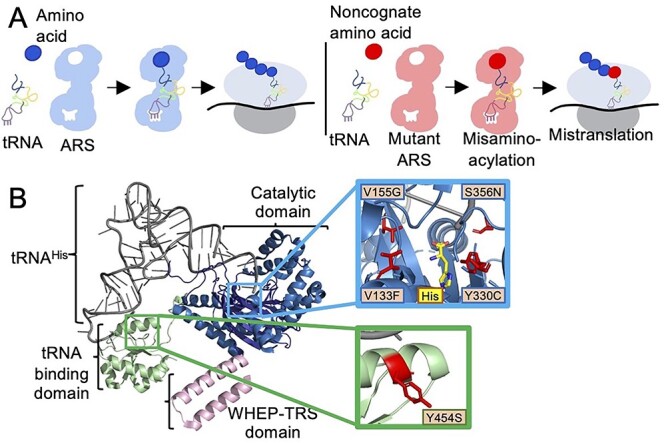
(**A**) Aminoacylation of tRNA by ARSs and mistranslation. Wild-type ARSs (blue) charge cognate amino acids onto tRNAs for protein translation. Mutations in ARSs (red) can lead to charging of non-cognate amino acids onto tRNAs, leading to mistranslation. (**B**) CMT2W and USH3B mutations in HARS. Monomer of human HARS (PDB Code: 6O76) with the catalytic domain (blue), anticodon-binding domain (green) and WHEP-TRS domain (pink), in complex with tRNA^His^. The blue square outlines a close-up view of the HARS active site with CMT2W associated mutations depicted as red sticks. Green square outlines the anticodon-binding domain with the USH3B associated HARS mutation Y454S depicted as red stick. Figure generated with PyMOL (Schrodinger, LLC).

Each ARS enzyme recognizes a specific amino acid and cognate tRNA to ensure high-fidelity translation of gene products (13). Mis-aminoacylation and potentially mistranslation can occur as a result of interactions between an ARS and a non-cognate amino acid or a non-cognate tRNA (Fig. 1A). ARSs distinguish their cognate tRNA from a large pool of similar tRNAs by the presence of identity element nucleotides (14). For most ARSs, an anticodon-binding domain directly reads one or more bases of the anticodon, physically linking the amino acid to its codon assignment. Identity elements are also found throughout the tRNA body, including the variable arm. Indeed, ARSs for serine and alanine do not recognize the anticodon at all, while HARS has a critical identity element outside of the canonical tRNA body. In a diversity of species including yeast and humans, HARS recognizes a unique G^−1^ residue, which in eukaryotes is added post-transcriptionally by tRNA^His^ guanylyltransferase (Thg1) (15). Although HARS does not recognize the tRNA^His^ anticodon, the Thg1 enzyme uses the tRNA^His^ anti-codon as its key recognition site to append the G_−1_ residue. A tRNA^His^ lacking the G^−1^ residue is not a competent substrate for HARS (16).

Human HARS is a dimeric protein and contains a WHEP-TRS domain, a catalytic domain and a tRNA binding domain (Fig. 1B). Aminoacylation and tRNA recognition functions have been studied in most detail in bacterial HARS (17–21), but several studies have investigated the functions of human HARS and the effects of pathogenic mutations. Autosomal dominant mutations in the catalytic domain of HARS have been associated with CMT2W (7,10,11,22,23), while mutations in the HARS anticodon-binding domain were associated with Usher syndrome type IIIB (USH3B), a disease of progressive hearing and vision loss (24). Although there is currently no cure for CMT, histidine supplementation has been explored as a treatment option for USH3B patients, with a clinical trial currently in its final phase (Clinicaltrials.gov NCT02924935).

We used *Saccharomyces cerevisiae* as a model organism to study human disease causing HARS mutations V133F, V155G, Y330C and S356N that are associated with CMT2W, and Y454S, which is associated with USH3B. We hypothesized that histidine supplementation would rescue growth defects in cells that rely on expression of disease-causing HARS variants. We found that human HARS mutations V133F, V155G, Y330C, S356N and Y454S can complement the deletion of *S. cerevisiae* yeast HARS ortholog HTS1, establishing a yeast model for CMT2W disease and USH3B disease. We show that all CMT2W mutations lead to significant growth deficiencies. We identified several mutation-specific phenotypes, including increased protein aggregation, and reduced HARS abundance and stability. We further show that histidine supplementation rescues the growth deficit caused by mutations V155G and S356N. Conversely, histidine caused synthetic toxicity with HARS V133F and Y330C. Our data indicate an allele-specific response to histidine supplementation in cells from different disease-linked HARS mutants. Our yeast model provides a rapid approach to screen HARS variants for their responsiveness to histidine. We anticipate that histidine will be a promising intervention for individuals suffering from CMT2W peripheral neuropathy if the activity of their HARS allele is rescuable by elevated histidine.

## Results

### Generation of a yeast model for HARS deficiency

**Figure 2:**
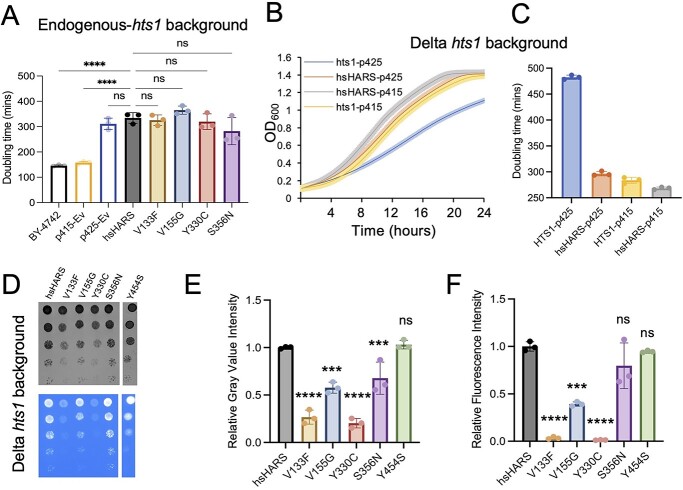
Dose-dependence of HARS complementation. (**A**) Doubling time of haploid wild-type yeast (BY-4742 with endogenous *hts1*) with plasmids expressing wild-type or mutant human HARS, or empty plasmids (Ev). Growth curve (**B**) and doubling time (**C**) of control strains hsHARS and *hts1* in low (p415) and high (p425) copy number plasmids. Yeast cultures were grown in SD Leu^−^ medium in 96-well plates for 24 h with 10-min read intervals with three biological replicates and three technical replicates each. BY-4742 is the yeast control strain without plasmids. One standard deviation represented by error bars. (**D**) Spotting assay at 30°C. Yeast strains were grown at 30°C in SD Leu^−^ medium, normalized to OD_600_ = 1 and serially diluted 1:1, 1:4, 1:4^2^, 1:4^3^ and 1:4^4^. Three replicates of plates were incubated in 30°C for 4 days. Spot plates were imaged using the Gel Doc XR+ (Biorad) under normal light (left) and UV light (right) to verify the expression of YFP fused to HARS. All CMT mutants grew slower, and V133F, V155G and Y330C showed less fluorescence than hsHARS. Quantification of spotting assay at 30°C for mean gray spot intensity (**E**) and fluorescence spot intensity (**F**). Spots were quantified using ImageJ as described (26). (^*^^*^^*^^*^P < 0.0001, ^*^^*^^*^P < 0.001, ns = statistically non-significant.)

To investigate the biochemical and cellular effects of HARS deficiency in cells, we generated a yeast model system. Since CMT mutants are known to cause a dominant phenotype in humans, where one mutated allele is sufficient to cause a disease phenotype, we first transformed haploid wild-type yeast with plasmid expressing wild-type or mutant human HARS. While the p425 vector used for high HARS expression increased doubling time compared to the wild-type haploid BY-4742 cells, we observed no additional effect on the yeast growth phenotype (Fig. 2A) due to HARS or mutant HARS expression. We therefore decided to proceed with a yeast model that relies entirely on human HARS activity to allow for a more defined model system (generation of model system: Supplementary Material, Fig. S1). We deleted endogenous yeast *hts1*, and expressed human HARS variants *in trans*. Interestingly, previous attempts to generate a model system for these HARS variants were not successful (10). Our approach (Supplementary Material, Fig. S1A) began with a diploid *hts1* heterozygous deletion strain with one essential *hts1* allele and one KanMX gene, obtained from the yeast haploid deletion collection (25). Human wild-type HARS (hsHARS) fused to yellow fluorescent protein (YFP) was cloned into p426-ccdB-GDP (URA3) yeast episomal plasmid, transformed into the parental diploid deletion strain, and successful transformation selected for on SD medium lacking uracil (SD Ura- (−)). Cells were sporulated and dissected (Supplementary Material, Fig. S1B), and five colonies (I–V) of haploid cells carrying human HARS and the expression of the KanMX gene (Supplementary Material, Fig. S1C) were selected on SD Ura^−^ plates (Supplementary Material, Fig. S1D) and geneticin (G418) plates (Supplementary Material, Fig. S1E). To confirm that colonies I–V were indeed haploid ∆*hts1* strains, we verified that (i) there was visually slower growth of colonies I–V compared to the corresponding diploid ∆*hts1* strain (Supplementary Material, Fig. S1F); (ii) colonies I-V mated only with MAT a tester strain suggesting that they were initially α haploids (Supplementary Material, Fig. S1G) and (iii) colonies I-V failed to survive when patched on a 5-fluoroorotic acid (5foa) plate, unlike the corresponding diploid strain (Supplementary Material, Fig. S1H). Thus, we confirmed that we have generated a haploid ∆*hts1* yeast model that is complemented by hsHARS to maintain survival.

Mutant HARS variants were introduced into the haploid deletion background by plasmid shuffling. The wild-type or mutant hsHARS variants (V133F, V155G, Y330C, S356N, Y454S point mutations) encoded in a p425-ccdB-GDP (LEU2) plasmid were transformed into the haploid ∆*hts1* strain containing the p426-HARS maintenance vector, and successful transformation was selected for on SD medium lacking uracil and leucine (SD Ura^−^ Leu^−^). These strains were grown on 5foa medium toxic to URA3^+^ cells (Supplementary Material, Fig. S1I), and successful plasmid shuffling was selected for on SD Leu^−^ medium for LEU2+ (Supplementary Material, Fig. S1L). No growth on SD Ura^−^ (Supplementary Material, Fig. S1J) or SD Ura^−^ Leu^−^ plates (Supplementary Material, Fig. S1K) verified successful plasmid shuffling, as well as the complementation of mutant HARS for the deletion of yeast HTS1. YFP fluorescence in cells confirmed successful HARS-YFP expression (Supplementary Material, Fig. S2). Thus, we successfully generated a model system to study HARS deficiency caused by five different HARS mutations in a ∆*hts1* yeast background (Fig. 2B and C).

### Dose-dependence of HARS complementation

To determine the impact of HARS expression level on cell growth, we investigated whether high (2 micron, p425 plasmids) or low (centromeric, p415 plasmids) copy number plasmids were able to complement our yeast HTS1 deletion strain. Both yeast HTS1 and wild-type hsHARS complement the yeast deletion strain (Fig. 2B and C). High doses of yeast HTS1 are increasingly toxic to yeast cells, whereas wild-type hsHARS is tolerated at high and low expression levels (Fig. 2C). The data show a dose-dependent phenotype for HTS1 complementation, suggesting that overactivity of yeast histidyl-tRNA synthetase creates proteotoxic stress in cells. No significant toxicity was observed for human HARS (Fig. 2B and C). Interestingly, our attempts to complement the deletion strain with any of the mutant hsHARS alleles using a low copy centromeric plasmid failed in agreement with previous studies (10). These data show that high levels of mutant human HARS are necessary and sufficient to sustain translation in a yeast HTS1 deletion strain and that all mutants except the Y454S HARS allele cause HARS deficiency manifesting in a growth phenotype.

### Mutations in the HARS active site cause global growth defects

**Figure 3:**
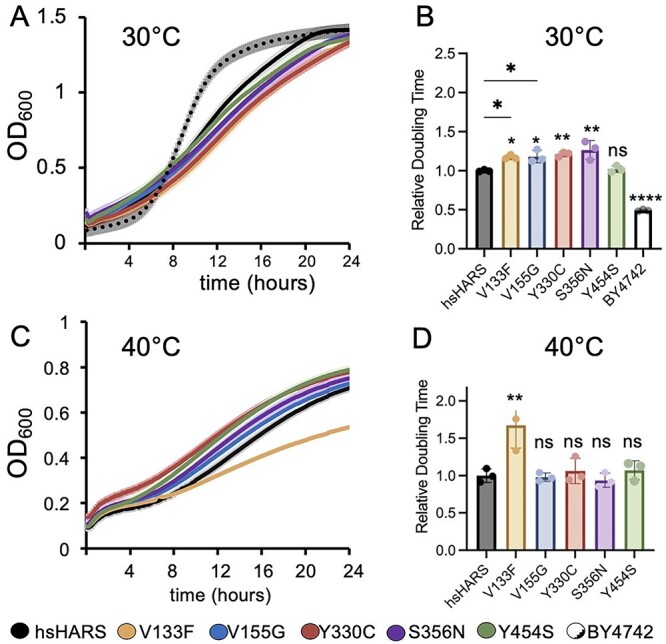
CMT-HARS leads to growth impairments. Growth curve at (**A**) 30°C and (**C**) 40°C. Yeast cultures were inoculated overnight in SD Leu^−^ medium until OD_600_ = 1, then incubated in Synergy-H1 plate reader (BioTek) for 24 h with 10-min read intervals using three biological replicates and three technical replicates per biological. BY-4742 is the yeast control strain without plasmids. One standard deviation of each data point represented by lighter coloured error bars. (**B**) Doubling time at 30°C and (**D**) 40°C. The doubling time was obtained from growth curves in A and B. (^*^^*^P < 0.01, ^*^P < 0.05, ns = statistically non-significant.)

To investigate the growth phenotype of the HARS yeast model, we performed spotting assays (Fig. 2D and E) and growth curve analysis at 30°C (Fig. 3A), as well as at 40°C, to induce heat stress (Fig. 3C). At 30°C, cells expressing the CMT2W associated mutants (V133F, V155G, Y330C and S356N) displayed significantly decreased growth and increased doubling times on solid and in liquid medium compared to hsHARS, while the Y454S mutant sustained wild-type-like growth (Figs 2D and E and 3B). Thus, all CMT-HARS mutants lead to growth impairments in the yeast model at 30°C. USH3B patients with HARS Y454S mutations show an increase in disease phenotype during febrile episodes, indicating that increased temperature exacerbates the effect of the HARS mutations. We therefore tested the growth phenotypes of the yeast models under heat stress. Interestingly, there was no significant difference in doubling time between the V155G, Y330C and S356N mutants compared to hsHARS. However, the V133F growth impairment was exacerbated at 40°C (Fig. 3D). Surprisingly, the Y454S mutant did not show a significant growth phenotype at 40°C (Fig. 3D), even though fever significantly escalates the disease phenotype of USH3B patients (27). These data show that HARS CMT2W associated mutants cause a significant growth defect in our yeast model, making yeast a suitable model organism for further characterization.

### HARS V155G and S356N induce protein aggregation in yeast

**Figure 4:**
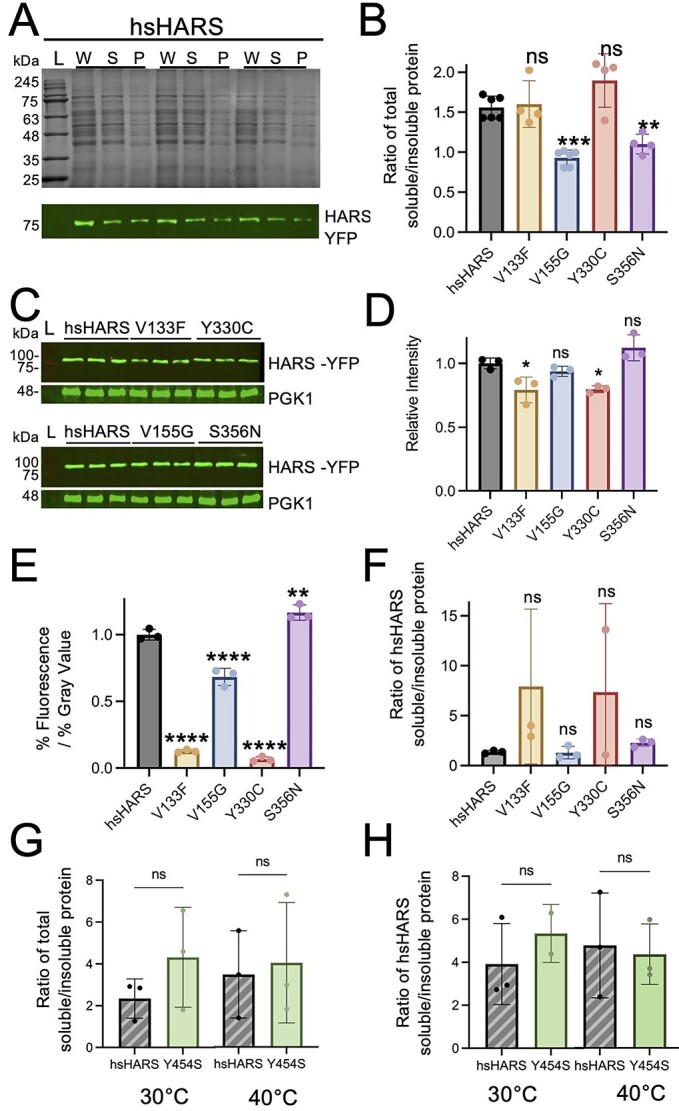
CMT-HARS mutants lead to global protein aggregation or reduced HARS expression. (**A**) SDS gel of hsHARS sedimentation assay. First lane (L) is the protein ladder. Equal volumes of whole lysate (W), soluble fraction (S) and insoluble fraction (P) portions extracted from different yeast variants in the sedimentation assay were loaded on to 15% SDS gel in three replicates. (**B** and **G**) Ratio of soluble to insoluble total protein fraction for CMT mutants (B) and Y454S mutant (G). Gels were quantified using ImageJ. A lower ratio represents a higher proportion of insoluble fraction compared to soluble fraction. (**C**) Western blot and (**D**) quantification of blots for HARS in whole lysate portion. Equal volume of whole lysate extracted from each yeast strain was loaded and blotted for HARS. Blots were imaged using the Li- Cor Odyssey 9120 imaging system. Western blot bands for HARS were quantified using ImageJ. (**E**) Ratio of %Fluorescence over %Gray value spot intensities. This ratio measures the expression of functional fluorescence-producing HARS-YFP fusion protein, relative to the amount of growth for each mutant. %Gray value is the relative intensity of spots between mutants and hsHARS in the spotting assay calculated in Fig. 2D. %Fluorescence is the YFP quantification from Fig. 2E. (**F**) Ratio of soluble to insoluble hsHARS-blotted fraction for CMT mutants (F) and Y454S mutant (**H**). Western blot of sedimentation assay gels using an anti-GFP antibody were imaged using the Li- Cor Odyssey 9120 imaging system and quantified using ImageJ. (^*^^*^^*^^*^P < 0.0001, ^*^^*^^*^P < 0.001, ^*^^*^P < 0.01, ^*^P < 0.05, ns = statistically non-significant.)

Misfolded proteins aggregate and accumulate in cells (28). To investigate whether the HARS mutations lead to protein misfolding and aggregation in yeast, we performed a sedimentation assay. The ratio of soluble to insoluble protein levels in cells expressing wild-type human HARS or mutant HARS was determined (Fig. 4A, Supplementary Material, Figs S3A–D and S4A). Interestingly, hsHARS V155G and S356N, but not V133F and Y330C, caused a significant increase in the insoluble protein fraction compared to wild-type hsHARS, indicating the accumulation of unfolded or misfolded proteins (Fig. 4B). In cells expressing mutant hsHARS, we observed an increase in the fraction of insoluble proteins compared to cells expressing wild-type hsHARS by 1.8-fold for V155G and 1.5-fold for S356N (Fig. 4B). The changes in protein solubility were evident across the proteome and not restricted to particular protein bands on the sodium dodecyl-sulfate polyacrylamide gel electrophoresis (SDS-PAGE) (Fig. 4A, Supplementary Material, Fig. S3A–D), indicating HARS alleles V155G and S356N lead to global protein misfolding in cells. For the Y454S variant, the assay was performed with cells grown at 30 and 40°C, yet we found no evidence of increased unfolded proteins in Y454S-expressing cells compared to cells expressing wild-type hsHARS (Fig. 4G).

### V133F and Y330C lead to reduced HARS abundance in yeast

A previous study showed that the USH3B mutant Y454S leads to reduced recombinant protein stability (24). We therefore investigated whether expression of mutant proteins in yeast leads to an increase of HARS proteins in the insoluble protein fraction. We first tested overall HARS abundance by western blotting whole cell lysates (Fig. 4C). The V133F and Y330C mutations lead to significantly reduced abundance of HARS in the whole lysate by 1.3-fold compared with hsHARS (Fig. 4D). The V155G and S356N variants were produced to a similar level as wild-type hsHARS. Since the HARS genes are each expressed from the same promoter on identical plasmids, wild-type and mutant HARS gene expression itself should not be affected by the mutations, but defects in HARS protein stability may lead to degradation of mutant HARS proteins.

To more accurately quantify HARS abundance in cells, we measured YFP fluorescence from the YFP-HARS fusion protein in living cells on spot plates (Fig. 2D). Three mutants led to significantly reduced YFP fluorescence intensity with reductions for V133F (29-fold), V155G (2.5-fold) and Y330C (76-fold) (Fig. 2F). Since several mutants displayed a reduced growth phenotype, we next calculated the ratio of fluorescence to gray value spot intensity to determine the level of HARS expression per cell (Fig. 4E). After accounting for differences in cell growth, we found that all mutants except for S356N displayed a significant reduction of fluorescence/spot intensity compared to wild-type hsHARS. V133F and Y330C mutants led to a 10-fold reduction in fluorescence per cell, whereas V155G (1.5-fold) showed more modest reductions in abundance in live cells (Fig. 4E). A reduction in fluorescence can indicate either reduced protein abundance, or indicate misfolded, non-fluorescent proteins (29).

To test whether HARS aggregation is responsible for the observed decrease in HARS-YFP fluorescence, we blotted for HARS in the total, soluble and insoluble protein fraction (Fig. 4A blot, Supplementary Material, Figs S3E and S4B). Interestingly, insoluble HARS does not significantly aggregate in the mutants compared to wild-type hsHARS expressing cells, as the ratio of HARS in the soluble and insoluble fraction remains unchanged in all mutants (Fig. 4F). For Y454S, which was previously shown to be temperature sensitive, we tested HARS protein aggregation at both 30°C and 40°C, but no increase in HARS in the insoluble fraction was observed (Fig. 4H). Together, these data show that the V133F and Y330C mutants lead to significant 10-fold reduction in HARS-YFP fluorescence in live cells, accompanied with a 1.25-fold reduced protein abundance as measured by western blotting (Fig. 4D and E), indicating HARS V133F and Y330C protein misfolding in yeast, leading to subsequent degradation. For V155G, the 1.5-fold reduced fluorescence/gray value was not accompanied with a significant change in protein abundance, and S356N shows minor (1.2-fold) increased fluorescence/gray value accompanied with no change in HARS abundance (Fig. 4D and E), indicating that HARS misfolding and degradation alone are not major contributors to the observed growth phenotype.

### Mutants V133F, V155G and Y454S lead to reduced HARS protein stability

**Figure 5:**
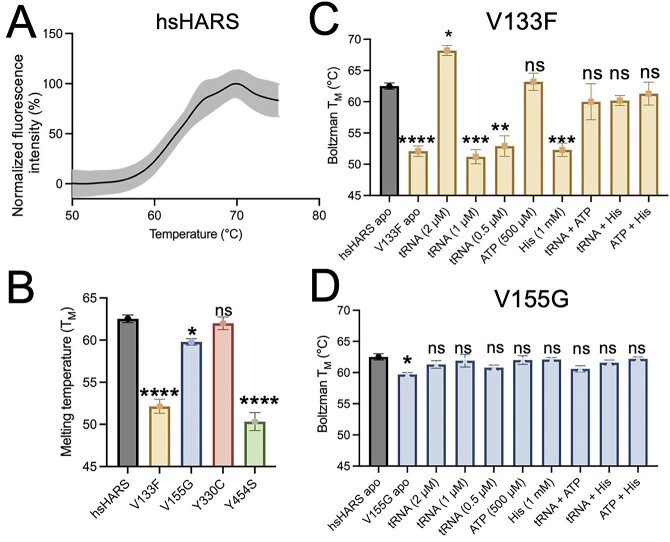
Recombinant proteins mutants V133F, V155G and Y454S lead to reduced protein stability, which can be rescued by substrate supplementation. (**A**) Melting curve of hsHARS obtained by differential scanning fluorimetry. Recombinant hsHARS, V133F, V155G and Y330C HARS protein were purified from *E. coli* and diluted to final concentration 1 μM. Samples were heated from 25 to 96°C, and fluorescence intensity was measured to follow unfolding of the proteins. Assays were carried out in quadruplicate, the standard error of each data point represented by lighter colored error bars. (**B**) T_M_ of HARS variants in apo form show reduced stability of V133F, V155G and Y454S. Initial analysis was performed in the Protein Thermal Shift™ software, and data were fit to the Boltzmann equation to estimate the melting temperature (T_M_). Bars indicate the mean of four replicates ± the standard error. T_M_ of (**C**) V133F and (**D**) V155G variants with substrate supplementation. Protein variants were incubated with buffer (apo HARS) and either 2 μM, 1 μM or 0.5 μM of tRNA^His^, 1 mM histidine or 500 μM ATP either on their own, in combination with each other, or with 2 μM tRNA^His^. (^*^^*^^*^^*^P < 0.0001, ^*^^*^^*^P < 0.001, ^*^^*^P < 0.01, ^*^P < 0.05, ns = statistically non-significant.)

To investigate the stability of mutant HARS proteins, we constructed and purified His-tagged hsHARS and variants from *Escherichia coli* (Supplementary Material, Fig. S5A). Purified proteins were analysed by differential scanning fluorimetry to measure protein stability and protein–ligand interactions. To quantify protein stability, the melting temperature (T_M_) (Fig. 5B) was determined from the melting curves of hsHARS (Fig. 5A) and mutant HARS variants (Supplementary Material, Fig. S5B–E). We were unable to purify sufficient protein of the S356N mutant, because the protein precipitated quickly and complicated the reproducibility of the experiments. We show that the melting temperature of Y454S is reduced from 62.5 ± 0.8°C (hsHARS) to 50 ± 1.8°C for the mutant protein. Similarly, we found a significant reduction in T_M_ for mutants V133F to 52 ± 1.2°C and V155G to 59.8 ± 0.7°C, demonstrating reduced protein stability. No significant change in stability was observed for the HARS Y330C protein (Fig. 5B, Supplementary Material, Fig. S5).

We next tested whether addition of the HARS substrate tRNA^His^_Mini_ at 2 μM, 1 μM, 0.5 μM, ATP at 500 μM, histidine at 1 mM, or a combination of substrates, would restore HARS stability. The tRNA^His^ Microhelix (tRNA^His^_Mini_) was previously shown as a suitable substrate for eukaryotic HARS (30–32). For wild-type hsHARS and HARS Y330C, tRNA, ATP or histidine supplementation had no effect on the T_M_ of the protein compared to apo form (Supplementary Material, Fig. S5G, Supplementary Material, Tables S3 and S4), indicating that substrate binding is not normally required for HARS protein stabilization. In the Y454S mutant, substrate supplementation led to a significant increase in T_M_ to a maximum of 58.6 ± 0.1°C for ATP in combination with histidine (24) (Supplementary Material, Fig. S5F, Supplementary Material, Table S5), but did not quite rescue stability to wild-type levels (62.5 ± 0.8°C) as described previously (24). The addition of tRNA or ATP alone or in combination slightly increased stability, while histidine supplementation drastically restored thermal stability (57.8 ± 0.8°C).

Interestingly, substrate supplementation restored thermal stability for both V133F (Fig. 5C, Supplementary Material, Table S6) and V155G (Fig. 5D, Supplementary Material, Table S7) to wild-type levels. For V133F, thermal stability was initially most drastically reduced by 10.4°C compared to wild-type HARS with a T_M_ = 52 ± 1.2°C. The addition of tRNA slightly but significantly increased thermal stability at lower tRNA concentrations, but at a high tRNA concentration restored thermal stability to above wild-type levels (T_M_ = 68 ± 1.1°C), indicating that tRNA binding can rescue the mutant HARS instability.

Similarly, high ATP concentrations increase the thermal stability of HARS V133F to wild-type levels (to T_M_ = 63 ± 1.9°C), while histidine addition has no effect on V133F. Thermal stability of V155F was reduced compared to wild-type (reduced by 2.7°C), and all substrate supplementations restored wild-type like levels of protein stability (Fig. 5D, Supplementary Material, Table S7). The thermal shift assay demonstrates that mutants V133F and Y454S are significantly less thermodynamically stable than hsHARS, and V155G is only slightly reduced and easily restored in stability. Y454S protein instability can be rescued directly by histidine supplementation, whereas V133F protein stability was rescued by high tRNA or ATP concentrations.

### Histidine supplementation rescues HARS V155G and S356N growth defects and prevents accumulation of insoluble proteins

**Figure 6:**
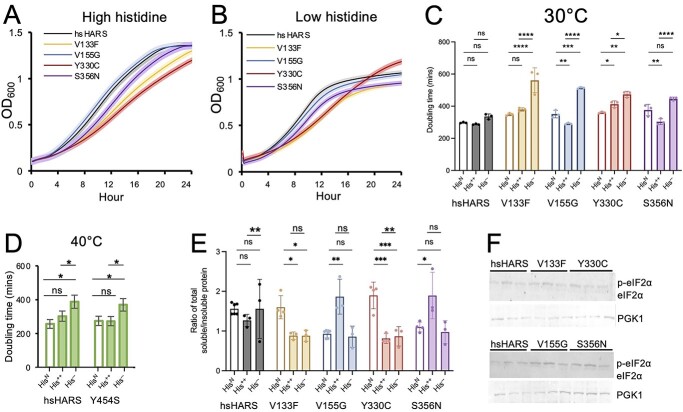
Histidine supplementation rescues V155G and S356N mutants’ growth deficiency and protein aggregation. Growth curve of yeast grown in (**A**) high histidine and (**B**) low histidine conditions. Yeast cultures were incubated in Synergy-H1 plate reader (BioTek) for 24 h in 30°C with 10-min read intervals using three biological replicates and three technical replicates per biological. For His^++^, histidine was supplemented to a final concentration of (200 mg/l) in the growth medium. For His^—^, histidine was reduced to 2 mg/l. Normal growth medium contains 20 mg/l histidine. One standard deviation of each data point represented by lighter-coloured error bars. Comparison of doubling time in high histidine (His^++^) and low histidine (His^—^) to normal histidine (His^N^) conditions for CMT mutants (**C**) and Y454S mutant (**D**). Doubling time obtained by calculating the slope of the exponential phase of the growth curve, and using the equation, doubling time = (log (2))/slope. (^*^^*^^*^^*^P < 0.0001, ^*^^*^^*^P < 0.001, ^*^^*^P < 0.01, ^*^P < 0.05, ns = statistically non-significant). (**E**) Ratio of soluble to insoluble protein fraction from a sedimentation assay for hsHARS and CMT mutant expressing cells grown under high and low histidine conditions. First lane (L) is the protein ladder. Equal volumes of whole lysate (W), soluble fraction (S) and insoluble fraction (P) portions extracted from different yeast variants in the sedimentation assay were loaded on to 15% SDS gel in three replicates. A lower ratio represents a higher proportion of insoluble fraction compared to soluble fraction. (**F**) Phostag gels probed for eIF2α showed predominantly phosphorylated eIF2α in mutant and wild-type human HARS expressing cells.

To investigate whether histidine substrate supplementation can rescue the growth of the yeast model, we performed a growth curve analysis of wild-type and mutant HARS under amino acid stress by increasing medium concentrations of histidine from 20 to 200 mg/l (Fig. 6A) or by reducing histidine levels in the growth medium (Fig. 6B) to 2 mg/l. Neither high nor low histidine growth conditions had a significant impact on yeast cells expressing wild-type hsHARS (Fig. 6, Supplementary Material, Table S8).

The four CMT2W associated mutants display two distinct phenotypes in response to the increased histidine concentration in the medium. While all CMT2W associated mutants show a significant increase in doubling time by 1.2–1.3-fold compared with wild-type hsHARS under normal growth conditions with 20 mg/l histidine (Fig. 3), this growth defect is amplified for the Y330C mutant when supplemented with histidine (Supplementary Material, Table S8, Fig. 6A and C). Cells expressing HARS Y330C showed an increased doubling time by 1.4-fold compared to normal histidine conditions. For mutants V155G and S356N, on the other hand, histidine supplementation showed a significant rescue effect and doubling times for cells expressing these variants at high histidine conditions were not significantly different from wild-type (Supplementary Material, Table S8, Fig. 6C). Cells expressing HARS V133F were not sensitive to increased histidine concentrations.

We next tested the effect of decreased histidine conditions on growth for the CMT2W associated mutants. Low histidine concentrations had no effect on wild-type or S356N expressing cells, while the three other mutants, V133F, V155G and Y330C, showed increased doubling times by ~1.5-fold (Fig. 6C). These results show that histidine supplementation can rescue the growth defect of HARS variants V155G and S356N, while conversely, histidine supplementation is toxic to Y330C expressing cells, suggesting that not all disease-causing HARS alleles will benefit from histidine supplementation.

To assess whether histidine supplementation resolves the accumulation of insoluble proteins in V155G and S356N mutants, we performed a sedimentation assay using cells grown under high histidine and low histidine growth conditions, while wild-type hsHARS expressing cells showed no change in insoluble protein accumulation (Fig. 6E, Supplementary Material, Fig. S7). For both V155G and S356N mutants, insoluble protein accumulation was rescued to wild-type levels under high histidine conditions, but not low histidine conditions, reflecting the rescued growth phenotype upon histidine supplementation. Interestingly, both increased and decreased histidine led to an increase of insoluble proteins in both V133F and Y330C mutants (Fig. 6E).

We and others show that Y454S reduces thermal stability, and the symptoms of USH3B HARS-related disease are exacerbated during febrile episodes (27). We therefore grew hsHARS and Y454S expressing yeast cells at elevated temperatures of 40°C, in low and high histidine conditions. Both Y454S and hsHARS carrying cells showed a generally decreased growth at 40°C (Supplementary Material, Fig. S6A and B). Increased histidine concentrations in the medium did lead to slightly increased lag phase for both hsHARS and Y454S, but no significant increase in doubling time was observed for either strain (Fig. 6D, Supplementary Material, Fig. S6C). On the other hand, decreased histidine availability led to a similar and significant decrease of growth in both strains, indicating that reduced histidine availability is disadvantageous under heat stress conditions (Fig. 6D, Supplementary Material, Fig. S6D). Overall, the Y454S mutation does not significantly increase doubling times compared to wild-type hsHARS at 30 or 40°C and did not lead to Y454S accumulation in the insoluble protein fraction (Figs 4G and 6D).

The accumulation of unfolded proteins was previously shown to induce the integrated stress response, which manifests in a phosphorylation of eukaryotic translation initiation factor 2α. We therefore probed for the phosphorylation of eIF2α, using a phostag-gel where phosphorylated and non-phosphorylated protein variants are separated due to a retardation of the phosphorylated variant (33). As described previously, p-eIF2α is the dominant form in *S. cerevisiae* (34,35), and we observed no change in phosphorylation status between wild-type and mutant HARS expressing form (Fig. 6F).

### HARS V155G and S356N, but not Y330C, cause mistranslation

**Figure 7:**
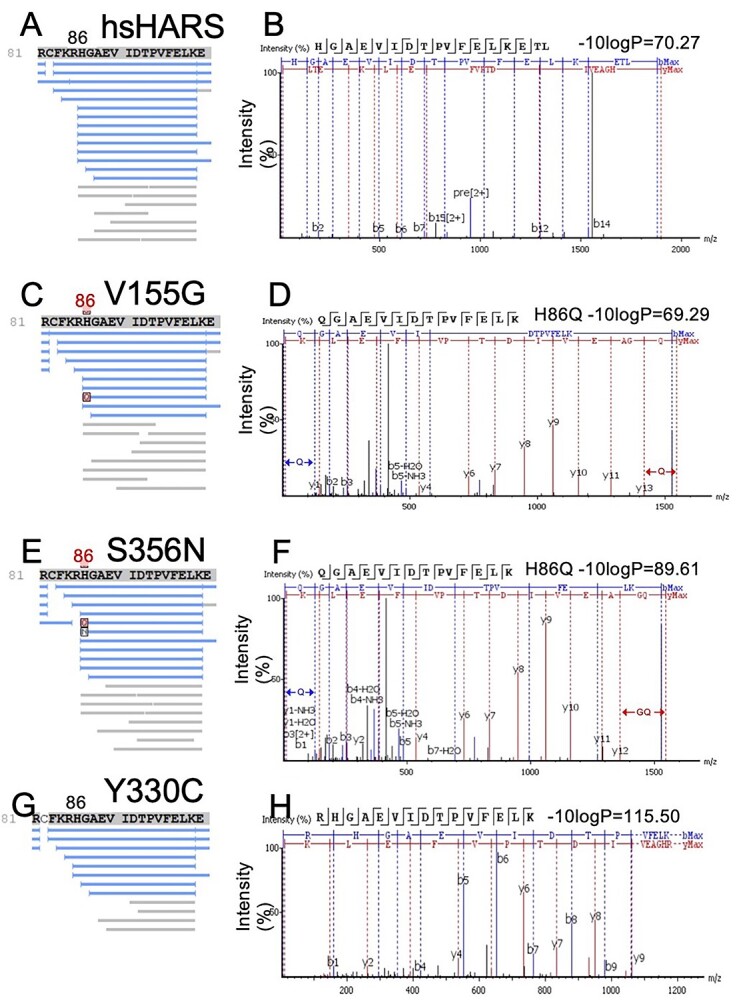
V155G and S356N cause mistranslation in yeast. Purified HARS-YFP expressed in hsHARS, V155G and S355N mutant yeast was in gel digested and submitted for mass spectrometry analysis at the Biological Mass Spectrometry Laboratory (The University of Western Ontario, London, Canada) to identify amino acids mis-incorporated in HARS-YFP. Protein coverage sequence of position 81–90 for (**A**) hsHARS, (**C**) V155G, (**E**) S355N and (**G**) Y330C yeast strains with mistranslation at H86Q highlighted. Corresponding peptide ion intensity spectra for (**B**) hsHARS, (**D**) V155G and (**F**) S355N yeast strains with –10logP scores annotated.

**Figure 8:**
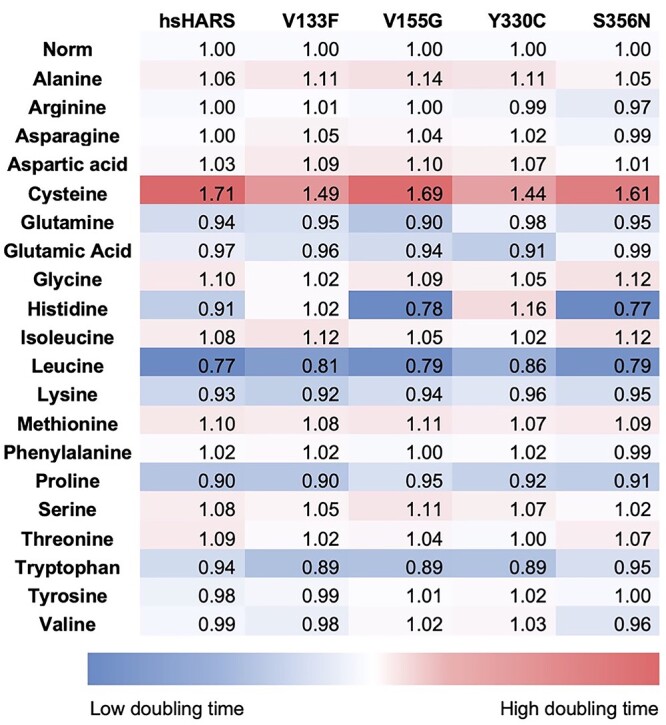
Amino acid stress does not significantly alter cell viability in mutant HARS expressing cells compared to wild-type cells. Yeast cultures were incubated in Synergy-H1 plate reader (BioTek) for 24 h in 30°C with 10-min read intervals using three biological replicates and three technical replicates per biological. Fold change in doubling times comparing 200 mg/l amino acid-supplemented conditions to normal conditions for hsHARS and CMT-HARS. Blue represents low fold change in doubling time, and red represents high fold change in doubling time.

The observed accumulation of unfolded proteins in V155G and S356N mutants in combination with the rescuing effect of histidine indicates a relaxed specificity of these HARS mutants that can be compensated with increased histidine availability, possibly displacing other amino acids from the HARS active site. This indicates misaminoacylation as a possible reason for mistranslation. We expected that the mutant HARS could aminoacylate tRNA^His^ with a non-cognate amino acid (ncAA), leading to ncAA-tRNA^His^ formation, and misincorporation of ncAA at histidine codons. Since HARS is expressed as a YFP fusion protein in our yeast model, we purified YFP-HARS from wild type, V155G and S356N mutants and used mass spectrometry to identify mistranslation events at histidine codons. In both mutant cell lines, we identified a signal for misincorporation of glutamine at position 86 (Fig. 7), with high peptide quality scores (69 for V155G and 89.6 for S356N). No H86Q peptide was found in wild-type or Y330C HARS expressing cells (Fig. 7A, B, G and H). We furthermore found mistranslation events at H553 to both threonine and glutamine. In S356N, we found two mistranslated H553T peptides (quality scores 73.39 and 70.35), as well as a H553Q (quality score 90.04) (Supplementary Material, Fig. S8C and D). In V155G, we identified two mistranslated H553T peptides (quality scores 61.21 and 73.21) (Supplementary Material, Fig. S8E and F). Interestingly, we also found a single H553E mistranslation event in cells expressing wild-type hsHARS (quality score: 64.31 for hsHARS) (Fig. 8A and B). Another mistranslation event at a histidine codon was observed at H553T in Y330C expressing cells (quality score: 72.7, Supplementary Material, Fig. S8G and H), indicating low or near background level mistranslation in Y330C, which does however not lead to insoluble protein aggregation. A H273Q peptide was identified for all four strains, albeit with higher quality scores and intensities in V155G and S356N (hsHARS: 68.7, V155G: 91.1, S356N: 101.8, Y330C: 75.9, Supplementary Material, Fig. S9). The mistranslation in wild-type HARS expressing cells is surprising, but a background level of mistranslation is known to occur in all cells (36). These data demonstrate that mutants V155G and S356N cause relaxed amino acid specificity above background levels and can cause misincorporation of threonine and glutamine, which can be rescued by favourably shifting the enzyme kinetics towards histidine.

### Amino acid stress does not exacerbate HARS deficiency

Since HARS mutants V155G and S356N lead to misincorporation of glutamine and threonine in response to histidine codons, we tested conditions for all the wild-type and mutant expressing HARS individually supplemented with each of the 20 amino acids. We generated growth curves and calculated doubling times (Supplementary Material, Table S9). Doubling times were then normalized to the doubling time of the corresponding strain in medium without additives, and a heat map was generated to display increased and decreased doubling times (Fig. 8). Many amino acids had no effect on the growth phenotypes, with few exemptions: As before, we show that histidine rescues V155G and S356N, but it exacerbates the effect of Y330C. Surprisingly, this was the only allele-specific phenotype we could identify. Cysteine negatively impacted the growth of all strains, showing cytotoxic effects in yeast, as shown before (37). Leucine, proline and tryptophan were generally favourable to growth phenotypes, but no significant difference was found between wild-type and mutant HARS variants. While we expected that glutamine or threonine addition may have a negative effect on V155G and S356N, this was not the case, indicating that glutamine and threonine restriction may not be necessary in potential patient treatment regiments.

## Discussion

Mutations in ARSs are often associated with diseases of the peripheral nervous system (PNS) (38). Several disease-causing HARS mutants were previously biochemically characterized. Mutations T132I, P134H, D175E, V244C, L305dup (duplication) and D364Y confer partial or complete loss of function in yeast (7), Y454S confers thermal lability in recombinant protein (24), while T132I, P134H and D175E lead to deficits in structural integrity of the HARS protein (11). In patient-derived samples, V133F leads to reduced aminoacylation activity (23) and Y454S to reduced histidine incorporation into the proteome (24). Mutations in other ARSs associated with peripheral neuropathy, however, retained wild-type like aminoacylation activity (4,39). It is unclear how and to what extent the loss of aminoacylation or stability in HARS cause diseases of the PNS, and no current cure or disease-specific treatment is available. Nutritional histidine supplementation is currently in a clinical trial for USH3B patients carrying a HARS Y454S mutation (ClinicalTrials.gov Identifier: NCT02924935). Since the impact of individual mutations on HARS function and disease progression are multifaceted and the disease-causing mechanism of each HARS mutant is not well understood, this apparently simple therapy may not work for all mutants.

To investigate the impact of histidine on pathological HARS variants in a cellular context, we designed a *S. cerevisiae* model system for CMT2W to study the impact of pathological HARS mutations on protein stability, the cellular proteome and cell viability. We phenotypically and biochemically characterized four HARS mutants associated with CMT2W (V133F, V155G, Y330C and S356N), as well as the USH3B associated mutation Y454S. In previous attempts to generate a yeast model system, mutations V155G, Y330C and S356N failed to complement the deletion of yeast HARS ortholog HTS1(10). We found that wild-type and mutant hsHARS are all able to complement the deletion of yeast HARS homolog HTS1*.* In agreement with previous studies, mutant hsHARS variants were unable to complement cells lacking HTS1 with low copy plasmids, but in higher copy number episomal plasmids, each of the hsHARS enable cell growth and viability. Thus, while the mutations impact HARS functionality in yeast, sufficient aminoacylation activity is retained for survival. Although V133F, V155G, Y330C and S356N were all located in the hsHARS active site, the mutants lead to different phenotypic responses in cells. Our data suggest that the mutants can be divided into two subcategories: V133F and Y330C mutations largely display a loss of aminoacylation, affecting cell growth. On the other hand, V155G and S356N mutations exhibit a gain-of-function effect, leading to global accumulation of insoluble proteins.

**Figure 9:**
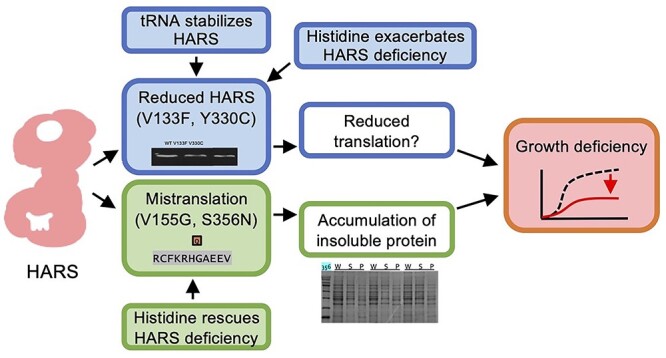
CMT2W associated mutations have different phenotypes. Mutants V133F and Y330C likely lead to reduced protein stability, which can be stabilized by tRNA. Histidine supplementation amplified the phenotype. Mutants V155G and S356N lead to reduced amino acid specificity, causing mistranslation and the accumulation of insoluble proteins. Histidine supplementation rescued this phenotype.

### HARS V133F and Y330C are loss-of function mutations

The CMT2W variant V133F was discovered in a single patient (23) and is linked to cognitive deficits and peripheral neuropathy. V133 is strictly conserved in eukaryotes, and the V133F mutant is associated with reduced aminoacylation activity in dermal fibroblasts, but overall protein synthesis and growth remains intact (23). Our data shows that V133F significantly reduced HARS thermal stability, which was rescued by either tRNA or ATP binding, but not histidine (Fig. 9). The Y330C mutation is localized opposite to V133F in the active site of HARS, and both amino acids coordinate the R-group of the histidine substrate (Fig. 1B). Y330C was identified in a cohort of patients (10) with autosomal dominant CMT disease. Recombinant Y330C shows reduced aminoacylation activity (10), and expression of Y330C in zebrafish disrupted the structure and function of the peripheral nervous system (40). Our data show that the Y330C mutation does not significantly affect thermal stability of recombinant HARS protein, which is in line with a previous observation that HARS Y330C was stably expressed in rat pheochromocytoma cells (PC12) (40). The small valine to bulky phenylalanine (V133F) substitution in the active site near the histidine aromatic residue leads to a drastic reduction of the active site binding pocket (Fig. 1B), and likely leads to significantly reduced binding of histidine and loss of structural integrity. The highly conserved aromatic tyrosine to small non-polar cysteine mutation (Y330C) could also disrupt amino acid binding and structural integrity, as Y330 is thought to lead to HARS stabilization upon histidine binding (41). Interestingly, we observed significantly reduced HARS abundance for both mutants in yeast cells (Fig. 9). It is therefore likely that V133F and Y330C lead to a loss-of-function disease phenotype, caused by reduced thermal stability or loss of aminoacylation. Interestingly, thermal instability of V133F can be rescued by tRNA^His^ and ATP, but not histidine, *in vitro.* Elevated histidine concentrations lead to the accumulation of insoluble proteins in both alleles and cause a further reduction in growth for V133F. HARS is one of the few synthetases that does not recognize the tRNA anticodon, but a unique G^−1^ residue at the acceptor stem near the active site (15). The thermal instability of V133F indicates a loss of the proteins’ structural integrity, potentially impeding the G^−1^ recognition. Future experiments will investigate if overexpression of tRNA^His^ may rescue V133F, which is stabilized by tRNA^His^  *in vitro*. A previous study showed that overexpression of tRNA^Gly^ rescued a mutant GARS enzyme that fails to release tRNA^Gly^, depleting the cellular tRNA^Gly^ pool and causing ribosome stalling on glycine codons (42). The exact role of elevated histidine in aggravating HARS deficiency and causing unfolded protein accumulation remains to be elucidated, but for now excludes histidine supplementation as a potential treatment for patients with V133F and Y330C mutations (Fig. 9). Balancing histidine requirements in Y330C or V133F mutation carrying CMT2W patients is therefore crucial to minimize the phenotypic effects of these pathogenetic HARS mutations.

### HARS V155G and S356N, but not Y330C disease variants cause protein mistranslation

Two HARS mutations, V155G and S356N, were associated with autosomal dominant CMT disease, with both enzymes displaying compromised aminoacylation activity *in vitro* (10). While our purified recombinant HARS S356N quickly aggregated, a previous study showed no significant reduction in thermal stability (10). Similarly, V155G was only slightly reduced in stability, and was rescued by histidine addition. Similar to studies in rat cells (40), we found HARS V155G is stably expressed in yeast. The V155G mutation was also studied in zebrafish, where V155G expression disrupts structure and function of the peripheral nervous system. Our data show that cells expressing either V155G or S356N showed reduced growth compared to wild-type expressing cells. We observed a significant increase in accumulation of insoluble proteins for both mutants and showed mistranslation of histidine to glutamine or threonine. Similarly, a study on threonyl-tRNA synthetase (TARS) mutants with reduced activity showed an increase in insoluble proteins (43,44).

There is currently no cure for CMT2W, yet histidine supplementation is a promising avenue for treatment of HARS-associated diseases. Excitingly, our data show both V155G and S356N cause a growth defect likely caused by insoluble protein accumulation in yeast that can be rescued to wild-type levels by histidine supplementation without restricting other amino acids (Fig. 9). Our data show that a high copy number plasmid is required to complement the yeast deletion strain with HARS mutants, yet wild-type hsHARS is sufficiently active to complement using a low copy plasmid, indicating reduced aminoacylation activity of the mutants. We also show that both mutations lead to mistranslation, global impaired translational fidelity, production of mutated and misfolded proteins, and finally unfolded protein accumulation. While it is possible that low His-tRNA^His^ availability may lead to near-cognate reading of histidine codons by e.g*.* Gln-tRNA^Gln^, the Y330C mutation did not lead to above background mistranslation or insoluble protein aggregation under normal histidine growth conditions, indicating that low His-tRNA^His^ levels alone do not necessarily lead to mistranslation. For V155G and S356N, the observed restoration of growth, and restoration of the proteome upon histidine supplementation shows that increased histidine outcompetes other amino acids in the active site of the mutant HARS proteins, leading to restored translational fidelity. In agreement with this data, low histidine concentrations increase the toxicity of V155G, perhaps because of mistranslation. These data indicate that V155G and S356N mutants lead to global mistranslation, which can be remedied by histidine supplementation.

### HARS Y454S associated with USH3B is rescued by histidine *in vitro*

A HARS c.1361A > C mutant was first detected in USH3B patients from the Old Amish Order in Lancaster County in Pennsylvania and a separate patient in Ontario (27). Subsequent biochemical experiments revealed no significant loss of aminoacylation, tRNA^His^ binding or dimerization of HARS. The HARS variants showed similar expression levels in fibroblast cells and similar intracellular localization compared to wild-type HARS (24,27). The pathogenicity of HARS Y454S is likely due to decreased thermal stability. A previous study showed that Y454S patient-derived fibroblast cells grown at elevated temperatures showed reduced protein synthesis compared to those of WT cells (24). Interestingly, we found that yeast cells expressing HARS Y454S did not show reduced growth compared to cells expressing wild-type hsHARS. As previously shown (24) and confirmed by our data, the Y454S mutant is less thermally stable than wild-type human HARS. We showed that the thermal instability of Y454S can be restored to almost wild-type levels by the addition of histidine, which provides a simple mechanism of action that supports histidine supplementation in USH3B patients. Consistent with the lack of a phenotype in yeast, no increase in protein misfolding was observed due to the Y454S mutation, and no significant growth defects were observed compared to cells expressing wild-type hsHARS under normal or conditions of histidine stress or deprivation.

The Y454S mutation is in the tRNA binding domain, at the catalytic domain interface. Y454S likely disrupts hydrogen bonding with catalytic domain residue E439, which may be the cause for the observed thermal instability. Rather than a loss of aminoacylation activity or histidine specificity, the HARS Y454S mutation may cause USH3B because of its thermal instability. The resulting loss of function and thermal instability can be rescued by histidine addition to facilitate efficient tRNA^His^ aminoacylation.

## Conclusions

Our data are directly relevant to ongoing clinical trials. Currently, L-histidine oral nutritional supplement to treat HARS deficiency in children with homozygous Y454S mutation is an approved clinical trial (NCT02924935). We developed a novel yeast model of CMT disease, which provided a rapid screening approach to evaluate human disease-causing HARS variants and determine the cellular and molecular consequences of histidine supplementation. Our study demonstrates that histidine supplementation can rescue mistranslating HARS mutants V155G and S356N, further supporting histidine supplementation as an avenue of treatment for these particular HARS variants. On the other hand, Y330C and V133F displayed synthetic toxicity with histidine, excluding histidine supplementation as a treatment. Amino acid supplementation should therefore only be considered after careful examination of biochemical, cellular and organismal effects dependent on the disease-causing allele.

## Materials and Methods

### Yeast strain and growth conditions

The *S. cerevisiae* ∆HTS1 heterozygous deletion strain was obtained from the *Saccharomyces* Genome Deletion Project (25), where one allele for *hts1* was replaced with a KanMX gene, leading to geneticin (G418) resistance. BY4742 is a haploid *S. cerevisiae* control strain. Cells were grown on Yeast Extract–Peptone–Dextrose (YPD) (10 g/l yeast extract, 20 g/l peptone and 20 g/l dextrose or 50 g/l of YPD Broth) or selective synthetic defined (SD) liquid medium (6.7 g/l yeast nitrogen base, 2% glucose, 60 mg/l L-isoleucine, 20 mg/l L-arginine, 40 mg/l L-lysine, 60 mg/l L-phenylalanine, 10 mg/l L-threonine, 10 mg/l L-methionine and 10 mg/l adenine hemisulfate salt), supplemented with 20 g/l agar for growth on solid medium. SD medium was supplemented with amino acids as adequate for the selectivity marker or the plasmid at 40 mg/l L-tryptophan, 20 mg/l L-histidine-monohydrate, 60 mg/l L-leucine or 20 mg/l uracil. Dissection YP plates were prepared with final concentrations of 10 g/l yeast extract, 20 g/l peptone, 2% glucose and 20 g/l agar. For growth curves, yeast cultures were grown in SD Leu^−^ medium in 96-well plates for 24 h. For spotting assays, yeast were spotted on SD Leu^−^ agar plates with 1:1, 1:4, 1:4^2^, 1:4^3^ and 1:4^4^ dilutions. For histidine assays, cells were grown with adjusted histidine concentrations for low histidine (2 mg/l) or high histidine (200 mg/l) at 30 or 40°C as indicated. For amino acid stress experiments, SD Leu^−^ medium was supplemented with 200 mg/l L-amino acids.

### Plasmids

HARS homolog in yeast (HTS1) was expressed from the pBY011 vector that was obtained from the DNASU plasmid repository in an *E. coli* DH5α stock (45). Wild-type hsHARS in pCAG/FLAG/RFC/A plasmid backbone was a kind gift from Dr Christopher S. Francklyn. All constructs were created through the standard procedures of Gateway cloning following the protocol developed by Invitrogen (46). p426-ccdB-GPD (URA3 plasmid) and p425-ccdB-GPD (LEU2 plasmid) are yeast episomal plasmids that stably propagate, which we used to express HARS as a HARS-YFP fusion. Site-directed mutagenesis primers were designed to generate the V133F, V155G, Y330C, S356N and Y454S mutations in HARS on the LEU2 plasmids as described previously (47). The entire open reading frames after Quickchange mutagenesis were sequenced. All plasmids are listed in Supplementary Material, Table S1. Primers are listed in Supplementary Material, Table S2.

### Yeast sporulation and dissection

p426-HARS-YFP plasmids were transformed into the diploid ∆HTS1 heterozygous deletion strain and sporulated as described (48). The spore colonies were genotyped by plating on SD plates lacking uracil to check for the expression of the 2-micron p426 plasmids and on YPD-G418 (350 μg/ml) to select for haploids containing the KanMX gene. Spores that grew on both the uracil-depleted and geneticin antibiotic G418-containing plates were (i) spotted on SD Ura (−) plates along with diploid ∆HTS1 cells for a diploid/haploid comparison, (ii) crossed with tester MATa and MATα haploid strains to ensure they were haploids and (iii) grown on 5foa plates to counter select for the p426 (URA3 expressing) plasmids.

### Plasmid shuffling

Growing cells harbouring a URA3 expressing plasmid (p426) on 5foa-containing plates obligates the cells to eliminate p426 in order to maintain survival (49). Following the transformation of LEU2 plasmids into the haploid ∆HTS1-HARS-p426 strain, the dual-plasmid transformants were grown on SD Leu (−) Ura (−) plates for 2 days at 30 °C. Single colonies were streaked on 5-foa agar plates for 3–5 days at 30 °C. Viable strains harbouring LEU2 plasmids only were selected and streaked on SD Leu (−), SD Ura (−) and SD Leu (−) Ura (−) plates for the verification of the plasmid shuffling. Strains that only grew on SD Leu (−) plates proceeded to further experiments.

### Yeast sedimentation assay and western blotting

The sedimentation assay was adapted from Shiber *et al*. (50). Briefly, yeast cultures are normalized to A_600_ = 1.0 and lysed. Cell lysis was fractioned into whole lysate, supernatant (soluble proteins) and pellet (insoluble proteins). To determine HARS expression, whole cell extracts were separated by SDS-PAGE and transferred to PVDF membrane (Roche Applied Science, Cat. No. 03 010 040 001). Anti-GFP antibody (Abcam, ab32146) was used at ratio of 1:5000. Secondary antibody IRDye® 800CW Goat anti-Rabbit IgG (Li-Cor, 926–32 211) was used at a ratio of 1:20 000 and detected using the Odyssey Classic (Li-Cor). Phostag gels were carried out as described previously (33) and probed with anti-eIF2α antibody (Invitrogen #AHO082).

### HARS purification

The hsHARS gene was integrated into a pDest527 destination vector using Gateway Cloning to yield a 6x-His tag expression construct. QuickChange site-directed mutagenesis methods were used to introduce point mutations to create V133F, V155G, Y330C, S356 and Y454S HARS variants. Plasmids encoding hsHARS variants were transformed into *E. coli* BL21 Codon Plus, and HARS proteins were purified following HisPur Ni-NTA column (Thermo Scientific #K95001) purification protocol according to the manufacturer’s instructions. All plasmids and primers are listed in Supplementary Material, Tables S1 and S2.

### Thermal shift assay

Differential scanning fluorimetry or thermal shift assay was performed as described previously (28). HARS protein samples were prepared on ice with a final concentration of 1 μM in a 96-well 0.1 ml microplate with 8X Protein Thermal Shift™ Dye and Protein Thermal Shift™ Buffer (ThermoFisher #4461146) to a final volume of 20 μl. tRNA^His^_Mini_ was used as tRNA (5’-GGCCAUCCUGCGGGGUGGCACCA-3′) that was previously shown to be a viable HARS substrate (32). Proteins were incubated with either 2 μM, 1 μM or 0.5 μM of tRNA^His^_Mini_. Samples were also incubated with 1 mM histidine or 500 μM ATP either on their own, in combination, or with 2 μM tRNA^His^_Mini_. Plates were sealed and protein unfolding was monitored with a QuantStudio™ 3 Real-Time PCR System (ThermoFisher). Samples were heated from 25 to 96°C at 1°C per minute, and fluorescence intensity was measured every 0.017°C. Initial analysis was performed in the Protein Thermal Shift software (ThermoFisher), and the data were fit to the Boltzmann equation to estimate the melting temperature (T_M_). Subsequent statistical analysis was performed through GraphPad Prism 9.1.0.

### Mass spectrometry

Yeast cells were lysed as previously described (50), and HARS-YFP was purified from cell lysates following the GFP-Trap Agarose kit protocol (Chromotek, Munich, Germany #gtak). Purified HARS-YFP was diluted with 2 × sodium dodecyl sulphate (SDS)-running buffer and separated using SDS-polyacrylamide gel electrophoresis (PAGE) with 12% acrylamide followed by Coomassie blue dye staining. The bands corresponding to HARS-YFP were identified at 84.4 kDa, excised from the gel, placed in sterile 1.5 ml centrifuge tubes containing 5% acetic acid and submitted for mass spectrometry analysis at the Biological Mass Spectrometry Laboratory (The University of Western Ontario, London, Canada). LysC was used to digest HARS-YFP and the QExactive Plus mass spectrometer (ThermoFisher) was used in FT/FT/HCD top 10 configuration to identify amino acids mis-incorporated in HARS-YFP as described before (29). The PEAKSX+ database was searched using a custom database, containing protein sequences of HARS-YFP with each of the mutations annotated in addition to a list of common contaminants.

## Statistical analysis

All statistical significance was obtained by running unpaired t-tests to compare the means and standard deviations between the control data set and the experiment data set or a one-way ANOVA test for multiple comparisons. Significance levels are indicated using asterisks (^*^^*^^*^^*^P < 0.0001, ^*^^*^^*^P < 0.001, ^*^^*^P < 0.01, ^*^P < 0.05, ns = statistically non-significant).

## Supplementary Material

Qiu_HMG_2022_Supplement_revision_ddac239

## Data Availability

The mass spectrometry data have been uploaded to the PRIDE database: PXD034865 (10.6019/PXD034865). Reviewer login details: Username: reviewer_pxd034865@ebi.ac.uk Password: uCXQxuR2.
