# Supplementary material for: Histidine supplementation can escalate or rescue HARS deficiency in a Charcot–Marie–Tooth disease model
Source: Hum Mol Genet. 2022 Sep 26;32(5):810–24. doi: 10.1093/hmg/ddac239 (PMC9941834; doi:10.1093/hmg/ddac239)
Supplement: Qiu_HMG_2022_Supplement_revision_ddac239 [file qiu_hmg_2022_supplement_revision_ddac239.doc]

**Histidine supplementation can escalate or rescue HARS deficiency in a Charcot Marie Tooth Disease model**

Yi Qiu^1#^, Rosan Kenana^1#^, Aruun Beharry^1#^, Sarah D.P. Wilhelm^1^, Sung Yuan Hsu^1^, Victoria M. Siu^1^, Martin Duennwald^2^, and Ilka U. Heinemann*^1^

^1^ Department of Biochemistry, The University of Western Ontario, London, Ontario, N6A 5C1, Canada

^2^ Department of Anatomy and Cell Biology, The University of Western Ontario, London, Ontario, N6A 5C1, Canada

^^ Authors contributed equally

* To whom correspondence should be addressed. Email: ilka.heinemann@uwo.ca

**Supplementary Tables 1-9 and Supplementary Figures 1-9.**

**Supplementary Table 1. Plasmids used in this study.**

| **Plasmids** | **Vector Backbone** | **Promoter** | **Replicon** | **Marker** | **Plasmid type** |
| --- | --- | --- | --- | --- | --- |
| *hts1*-p426 | pRS426 | GPD | 2 micron - Yeast Episomal plasmids (Yep) | URA3, ampR | Destination vector for native protein expression |
| hsHARS-p426 |  |  |  |  |  |
| Y454S-HARS-p426 |  |  |  |  |  |
| hsHARS-p426-YFP |  |  |  |  | Destination vector for fusion protein expression |
| Y454S-HARS-p426-YFP |  |  |  |  |  |
| *hts1*-p425 | pRS425 | GPD | 2 micron - Yeast Episomdeal plasmids (Yep) | LEU2, ampR | Destination vector for native protein expression |
| hsHARS-p425 |  |  |  |  |  |
| Y454S-HARS-p425 |  |  |  |  |  |
| hsHARS-p425-YFP |  |  |  |  | Destination vector for fusion protein expression |
| V133F-HARS-p425-YFP |  |  |  |  |  |
| V155G-HARS-p425-YFP |  |  |  |  |  |
| Y330C-HARS-p425-YFP |  |  |  |  |  |
| S356N-HARS-p425-YFP |  |  |  |  |  |
| *hts1*-p415 | pRS415 | GPD | CEN6 - yeast centromere plasmid (YCp) | LEU2, ampR | Destination vector for native protein expression |

**Supplementary Table 2. Primers used in this study.**

| **Primer ID** | **Primer Sequence (5’-3’)** | **Primer Direction** | **Purpose of use** |
| --- | --- | --- | --- |
| HARS_H_F | GGGGACAAGTTTGTACAAAAAAGCAGGCTCCATGGCAGAGCGTGCGGCG | Forward | Gateway cloning PCR to insert HARS into the system |
| HARS_H_stop_R | GGGGACCACTTTGTACAAGAAAGCTGGGTCTCAGCAGATGCAGAGGGGCT | Reverse |
| HARS_H_nonstop_R | GGGGACCACTTTGTACAAGAAAGCTGGGTcGCAGATGCAGAGGGGCT | Reverse |
| HARS_QKCHNG_c.1361C>A_F | GCTACTGAACCAGTTACAGTACTGTGAGGAGGCAGGCATCC | Forward | Quick  change PCR: from Y454S to hsHARS |
| HARS_QKCHNG_c.1361C>A_R | GGATGCCTGCCTCCTCACAGTACTGTAACTGGTTCAGTAGC | Reverse |
| HARS_QKCHNG_c.1361A>C_F | GCTACTGAACCAGTTACAGTCCTGTGAGGAGGCAGGCATCC | Forward | Quick  change PCR: from hsHARS to Y454S-HARS |
| HARS_QKCHNG_c.1361A>C_R | GGATGCCTGCCTCCTCACAGGACTGTAACTGGTTCAGTAGC | Reverse |
| HARS_QKCHNG_c.397G>T_F | CCTTCGCTATGACCTCACTTTTCCTTTTGCTCGGTATTT | Forward | Quick  change PCR: from hsHARS to V133F HARS |
| HARS_QKCHNG_c.397G>T_R | AAATACCGAGCAAAAGGAAAAGTGAGGTCATAGCGAAGG | Reverse |
| HARS_QKCHNG_c.464T>G_F | CGCTACCACATAGCAAAGGGATATCGGCGGGATAACCCA | Forward | Quick  change PCR: from hsHARS to V155G HARS |
| HARS_QKCHNG_c.464T>G_R | TGGGTTATCCCGCCGATATCCCTTTGCTATGTGGTAGCG | Reverse |
| HARS_QKCHNG_c.989A>G_F | CTTGCTCGAGGGCTGGATTGCTACACTGGGGTGATCTAT | Forward | Quick  change PCR: from hsHARS to Y330C HARS |
| HARS_QKCHNG_c.989A>G_R | ATAGATCACCCCAGTGTAGCAATCCAGCCCTCGAGCAAG | Reverse |
| HARS_QKCHNG_c.1067G>A_F | CTGGGTGTGGGCAATGTGGCTGCTGGA | Forward | Quick  change PCR: from hsHARS to S356N HARS |
| HARS_QKCHNG_c.1067G>A_R | TCCAGCAGCCACATTGCCCACACCCAG | Reverse |
| pDONR201_F | TCGCGTTAACGCTAGCATGGATCTC | Forward | Sequencing any gene in a pDONR201 vector |
| pDONR201_R | GTAACATCAGAGATTTTGAGACAC | Reverse |
| GPD-F | CGGTAGGTATTGATTGTAATTCTG | Forward | Sequencing any gene in p426, p425, p415 vectors |
| pBluescript KS_R | CTCGAGGTCGACGGTATCG | Reverse |
| HARS_seq_F1 | CGATACCGGGAATTCTACCA | Forward | Sequencing HARS to confirm mutagenesis |
| HARS_seq_F2 | AGGCAGTGCTGCTACAGACC | Forward |
| HARS_seq_F3 | CCCAAGGGCACAAGAGACTA | Forward | Sequencing HARS to confirm mutagenesis |
| HARS_seq_F4 | GCTGCTCCAGGATCCTAAACT | Forward | Sequencing HARS to confirm mutagenesis |
| HARS_F3 | CTCAGAACTGTGGGATGCTG | Forward | Sequencing upstream of HARS |
| HARS_R1 | TGAAGCAACGGATGATTACG | Reverse | Sequencing downstream of HARS |
| YFP_F1 | CGACCACTACCAGCAGAACA | Forward | To confirm YFP is on plasmid |
| HTS1_F | ATCTTCCAAATCGCTGGTGT | Forward | Sequencing *hts1* |
| HTS1_R | CGATAGTGACACCACCATGC | Reverse |

**Supplementary Table 3. Melting temperatures (T_M_) for wildtype hsHARS with and without substrate addition as determined by differential scanning fluorimetry**

| **hsHARS** | **T_M_** |
| --- | --- |
| T_M_ of apoenzyme: | 62.5 ± 0.8 |
| T_M_ with tRNA^His^_Mini_ (2 μM) | 61.9 ± 0.4 |
| T_M_ with tRNA^His^_Mini_ (1 μM) | 62.1 ± 1.4 |
| T_M_ with tRNA^His^_Mini_ (0.5 μM) | 62.0 ± 1.5 |
| T_M_ with ATP (0.5 mM) | 62.4 ± 0.4 |
| T_M_ with His (1 mM) | 62.9 ± 2.2 |
| T_M_ with tRNA^His^_Mini_ (2 μM) and ATP (0.5 mM) | 62.8 ± 0.1 |
| T_M_ with tRNA^His^_Mini_ (2 μM) and His (1 mM) | 63.0 ± 0.5 |
| T_M_ with ATP (0.5 mM) and His (1 mM) | 62.4 ± 0.6 |

**Supplementary Table 4. Melting temperatures (T_M_) for HARS Y330C with and without substrate addition as determined by differential scanning fluorimetry**

| **Y330C** | **T_M_** | **Fold Change to hsHARS** | **Significance vs. matching hsHARS condition** | **Significance vs. hsHARS** |
| --- | --- | --- | --- | --- |
| T_M_ of apoenzyme: | 62.0 ± 1.3 | 0.99 | n.s. | n.s. |
| T_M_ with tRNA^His^_Mini_ (2 μM) | 62.6 ± 2.2 | 1.00 | n.s. | n.s. |
| T_M_ with tRNA^His^_Mini_ (1 μM) | 62.8 ± 0.4 | 1.00 | n.s. | n.s. |
| T_M_ with tRNA^His^_Mini_ (0.5 μM) | 62.1 ± 1.4 | 0.99 | n.s. | n.s. |
| T_M_ with ATP (0.5 mM) | 63.1 ± 0.5 | 1.01 | n.s. | n.s. |
| T_M_ with His (1 mM) | 63.3 ± 0.7 | 1.01 | n.s. | n.s. |
| T_M_ with tRNA^His^_Mini_ (2 μM) and ATP (0.5 mM) | 62.5 ± 0.9 | 1.00 | n.s. | n.s. |
| T_M_ with tRNA^His^_Mini_ (2 μM) and His (1 mM) | 64.4 ± 1.8 | 1.03 | n.s. | n.s. |
| T_M_ with ATP (0.5 mM) and His (1 mM) | 61.6 ± 1.6 | 0.99 | n.s. | n.s. |

**Supplementary Table 5. Melting temperatures (T_M_) for HARS Y454S with and without substrate addition as determined by differential scanning fluorimetry**

| **Y454S** | **T_M_** | **Fold Change to hsHARS** | **Significance vs. matching hsHARS condition** | **Significance vs. hsHARS** |
| --- | --- | --- | --- | --- |
| T_M_ of apo enzyme | 50.3 ± 1.8 | 0.80 | p < 0.0001 | <0.0001 |
| T_M_ with tRNA^His^_Mini_ (2 μM) | 53.6 ± 0.7 | 0.86 | p < 0.0001 | <0.0001 |
| T_M_ with tRNA^His^_Mini_ (1 μM) | 54.3 ± 1.0 | 0.87 | p < 0.0001 | <0.0001 |
| T_M_ with tRNA^His^_Mini_ (0.5 μM) | 53.7 ± 1.2 | 0.86 | p < 0.0001 | <0.0001 |
| T_M_ with ATP (0.5 mM) | 53.7 ± 0.5 | 0.86 | p < 0.0001 | <0.0001 |
| T_M_ with His (1 mM) | 57.8 ± 0.8 | 0.92 | p < 0.0001 | <0.0001 |
| T_M_ with tRNA^His^_Mini_ (2 μM) and ATP (0.5 mM) | 53.1 ± 1.5 | 0.85 | p < 0.0001 | 0.0002 |
| T_M_ with tRNA^His^_Mini_ (2 μM) and His (1 mM) | 58.0 ± 0.3 | 0.93 | p < 0.0001 | 0.0003 |
| T_M_ with ATP (0.5 mM) and His (1 mM) | 58.6 ± 0.1 | 0.94 | p < 0.0001 | 0.0022 |

**Supplementary Table 6. Melting temperatures (T_M_) for HARS V133F with and without substrate addition as determined by differential scanning fluorimetry**

| **V133F** | **T_M_** | **Fold Change to hsHARS** | **Significance vs. matching hsHARS condition** | **Significance vs. hsHARS Apo form** |
| --- | --- | --- | --- | --- |
| T_M_ of apoenzyme: | 52.1 ± 1.2 | 0.83 | <0.0001 | 0.0007 |
| T_M_ with tRNA^His^_Mini_ (2 μM) | 68.2 ± 1.1 | 1.09 | <0.0001 | 0.0466 |
| T_M_ with tRNA^His^_Mini_ (1 μM) | 51.2 ± 1.6 | 0.82 | <0.0001 | 0.0003 |
| T_M_ with tRNA^His^_Mini_ (0.5 μM) | 52.9 ± 2.3 | 0.85 | <0.0001 | 0.0017 |
| T_M_ with ATP (0.5 mM) | 63.2 ± 1.9 | 1.01 | n.s. | ns |
| T_M_ with His (1 mM) | 52.3 ± 1.5 | 0.84 | <0.0001 | 0.0009 |
| T_M_ with tRNA^His^_Mini_ (2 μM) and ATP (0.5 mM) | 60.0 ± 4.1 | 0.96 | ns | ns |
| T_M_ with tRNA^His^_Mini_ (2 μM) and His (1 mM) | 60.2 ± 1.1 | 0.96 | ns | ns |
| T_M_ with ATP (0.5 mM) and His (1 mM) | 61.3 ± 2.6 | 0.98 | ns | ns |

**Supplementary Table 7. Melting temperatures (T_M_) for HARS V155G with and without substrate addition as determined by differential scanning fluorimetry**

| **V155G** | **T_M_** | **Fold Change to hsHARS** | **Significance vs. matching hsHARS condition** | **Significance vs. hsHARS Apo form** |
| --- | --- | --- | --- | --- |
| T_M_ of apoenzyme: | 59.8 ± 0.7 | 0.96 | p = 0.0279 | p = 0.00279 |
| T_M_ with tRNA^His^_Mini_ (2 μM) | 61.3 ± 1.1 | 0.98 | n.s. | n.s. |
| T_M_ with tRNA^His^_Mini_ (1 μM) | 61.9 ± 0.4 | 0.99 | n.s. | n.s. |
| T_M_ with tRNA^His^_Mini_ (0.5 μM) | 60.8 ± 0.8 | 0.97 | n.s. | n.s. |
| T_M_ with ATP (0.5 mM) | 62.0 ± 1.2 | 0.99 | n.s. | n.s. |
| T_M_ with His (1 mM) | 62.2 ± 0.7 | 0.99 | n.s. | n.s. |
| T_M_ with tRNA^His^_Mini_ (2 μM) and ATP (0.5 mM) | 60.7 ± 0.9 | 0.97 | n.s. | n.s. |
| T_M_ with tRNA^His^_Mini_ (2 μM) and His (1 mM) | 61.6 ± 0.8 | 0.99 | n.s. | n.s. |
| T_M_ with ATP (0.5 mM) and His (1 mM) | 62.3 ± 0.5 | 1.00 | n.s. | n.s. |

**Supplementary Table 8. Doubling time of yeast grown in high histidine and low histidine conditions.** (****p<0.0001, ***p<0.001, **p<0.01, *p<0.05, ns = statistically non-significant). Statistics relate to the comparison with wildtype hsHARS.

|  | High His | | | Low His | | |
| --- | --- | --- | --- | --- | --- | --- |
|  | Doubling time (mins) | Fold change to hsHARS | Significance compared to wild- type hsHARS | Low His | Fold change to hsHARS | Significance compared to wildtype hsHARS |
| *hsHARS* | *285 ± 3* | *-* | *-* | *350 ± 13* | *-* | *-* |
|  | *292 ± 3* | *-* | *-* | *340 ± 13* | *-* | *-* |
|  | *285 ± 3* | *-* | *-* | *320 ± 13* | *-* | *-* |
| V133F | 382 ± 9 | 1.3 | **** | 610 ± 63 | 1.7 | **** |
|  | 367 ± 9 | 1.2 | **** | 470 ± 63 | 1.4 | **** |
|  | 389 ± 9 | 1.4 | **** | 600 ± 63 | 1.9 | **** |
| V155G | 286 ± 4 | 1.0 | ns | 514 ± 4 | 1.5 | *** |
|  | 296 ± 4 | 1.0 | ns | 508 ± 4 | 1.5 | *** |
|  | 290 ± 4 | 1.0 | ns | 518 ± 4 | 1.6 | *** |
| Y330C | 390 ± 17 | 1.4 | **** | 450 ± 15 | 1.3 | ** |
|  | 420 ± 17 | 1.4 | **** | 480 ± 15 | 1.4 | ** |
|  | 430 ± 17 | 1.5 | **** | 490 ± 15 | 1.5 | ** |
| S356N | 300 ± 15 | 1.0 | ns | 450 ± 8 | 1.3 | * |
|  | 290 ± 15 | 1.0 | ns | 435 ± 8 | 1.3 | * |
|  | 320 ± 15 | 1.1 | ns | 452 ± 8 | 1.4 | * |

**Supplementary Table 9. Doubling time of yeast supplemented with 200 mg/L L-amino acids.** Norm represents normal growth media without amino acid supplementation. Doubling time in minutes.

|  | **hsHARS** | **V133F** | **V155G** | **Y330C** | **S356N** |
| --- | --- | --- | --- | --- | --- |
| **Norm** | 243 ± 5 | 324 ± 10 | 264 ± 5 | 354 ± 15 | 263 ± 11 |
| **Alanine** | 258 ± 7 | 359 ± 11 | 300 ± 4 | 394 ± 12 | 276 ± 8 |
| **Arginine** | 242 ± 1 | 326 ± 5 | 263 ± 5 | 351 ± 6 | 255 ± 2 |
| **Asparagine** | 243 ± 4 | 343 ± 5 | 273 ± 5 | 358 ± 9 | 252 ± 3 |
| **Aspartic acid** | 249 ± 2 | 356 ± 5 | 290 ± 7 | 375 ± 9 | 259 ± 5 |
| **Cysteine** | 425 ± 25 | 530 ± 14 | 491 ± 1 | 539 ± 23 | 416 ± 45 |
| **Glutamine** | 235 ± 2 | 338 ± 11 | 260 ± 6 | 369 ± 39 | 246 ± 3 |
| **Glutamic Acid** | 228 ± 2 | 324 ± 5 | 245 ± 3 | 336 ± 13 | 244 ± 4 |
| **Glycine** | 257 ± 5 | 345 ± 9 | 283 ± 5 | 389 ± 17 | 277 ± 10 |
| **Histidine** | 234 ± 4 | 353 ± 23 | 219 ± 10 | 452 ± 75 | 214 ± 2 |
| **Isoleucine** | 279 ± 9 | 388 ± 15 | 297 ± 5 | 399 ± 26 | 311 ± 18 |
| **Leucine** | 216 ± 8 | 315 ± 8 | 234 ± 10 | 341 ± 20 | 246 ± 12 |
| **Lysine** | 260 ± 7 | 355 ± 8 | 280 ± 13 | 383 ± 27 | 294 ± 17 |
| **Methionine** | 287 ± 12 | 384 ± 13 | 310 ± 16 | 412 ± 27 | 321 ± 17 |
| **Phenylalanine** | 264 ± 8 | 362 ± 9 | 280 ± 9 | 389 ± 29 | 292 ± 16 |
| **Proline** | 236 ± 3 | 325 ± 20 | 265 ± 4 | 358 ± 14 | 266 ± 11 |
| **Serine** | 284 ± 27 | 379 ± 14 | 310 ± 4 | 415 ± 15 | 299 ± 16 |
| **Threonine** | 310 ± 6 | 386 ± 11 | 321 ± 5 | 415 ± 8 | 320 ± 12 |
| **Tryptophan** | 266 ± 11 | 336 ± 12 | 276 ± 4 | 370 ± 14 | 283 ± 3 |
| **Tyrosine** | 261 ± 7 | 333 ± 8 | 278 ± 4 | 377 ± 12 | 281 ± 6 |
| **Valine** | 264 ± 23 | 328 ± 5 | 282 ± 16 | 382 ± 11 | 271 ± 11 |

**Supplementary Figure 1. (A)** **Workflow for the generation of HARS mutant model system in *S. cerevisiae***. p426 (URA3) plasmids carrying HARS-YFP is transformed into heterozygous diploids with only one functional *hts1* allele. Yeast sporulation and dissection yields haploid yeast colonies with no genomic *hts1* allele and are dependent on complementation by human HARS. Transformation of p425 LEU2 plasmids carrying wildtype HARS-YPF gene or HARS-YFP with point mutations (V133F, V155G, Y330C, Y356N, Y454S) yields dual plasmid haploids. The URA3 plasmid is subsequently eliminated, generating the yeast model for HARS deficiency. **(B)** Visible yeast tetrads from sporulation plates under the light microscope. (**C)** Dissection of tetrads showed smaller colonies (I-V) which were suspected to be the knockout haploids. All colonies on the sporulation plates were then patched on (**D)** SD Ura^-^ and **(E)** YPD-G418 plates. Five colonies labelled I to V survived on both plates and were selected for further experiments. Here, we show only colonies I-III on SD Ura^-^ and YPD-G418 plates. **(F)** **Spotting plates of post-dissection ∆*hts1* haploid selected colonies in comparison to a corresponding diploid.** Colonies A to E grow slower than their mother diploid strain bearing the same HARS-plasmid. Collected colonies A to E spotted on SD Ura^-^ plates imaged using the Gel Doc XR+ (Biorad) under normal light and UV light. **(G)** **Mating of strains A to E with Mat a or Mat α tester strains.** Colonies A to E mated only with Mat a tester strain and not Mat α suggesting that they were initially α haploids. **(H)** **Counter selection for URA3-expressing plasmids on 5foa plates.** Colonies A to E were patched on a 5foa-containing plate along with the parental ∆hts1 diploid strain expressing the same HARS plasmid (Y454S-HARS-YFP-p426) as a positive control. Colonies A-E failed to grow, thus, they were dependent on the introduced HARS-bearing plasmids for their survival. **(I)** **Plasmid shuffling.** Haploid yeast strains with both URA3-resistant plasmid carrying hsHARS and LEU2-resistant plasmid carrying *hts1*, hsHARS, or CMT-HARS were patched on 5foa plate for plasmid shuffling to kick out the URA3 plasmid. BY4742, haploid control yeast strain. *hts1*, yeast homolog of human HARS. Empty vector, negative control. **Yeast complementation negative control (J) SD Ura^-^ and (K) SD Ura^-^ Leu^-^ plates.** After plasmid shuffling, no growth on these negative control plates demonstrates that only the LEU2 plasmid remains. **(L) Successful selection of Yeast disease models.** Colonies after plasmid shuffling were streaked on SD Leu^-^ and incubated at 30°C for 4 days, showing successful complementation of haploid yeast *hts1* deletion cells.

**Supplementary Figure 2.** **Fluorescence microscopy of mutant HARS yeast model *in vivo*.** Wildtype (hsHARS), V133F, V155G, Y330C, S356N, and Y454S-*HARS* expressing strains were imaged under the EVOS M5000 microscope (Thermo Fisher) enabling visualization of HARS-YFP expression at 40X magnification.

**Supplementary Figure 3. Sedimentation assay SDS gels and Blots of (A)** **V133F, (B)V155G, (C)Y330C, and (D) S356N** First lane is the protein ladder (FroggaBio) with labelled molecular weights. Equal volumes of whole lysate (W), soluble fraction (S) and insoluble fraction (P) extracted from different yeast variants in the sedimentation assay was loaded on to 15% SDS gel in 3 replicates. Gels imaged using the Gel Doc XR+ (Biorad). **(E) Western Blots for HARS.** Western Blot of sedimentation assay gels performed using primary anti-GFP rabbit monoclonal (ab32146) in 1:10000 dilution, and secondary antibody used was IRDye® 800CW Goat anti-Rabbit IgG (926-32211) in 1:10000 dilution. Blots were imaged using the Li- Cor Odyssey 9120 imaging system and quantified using ImageJ.

**Supplementary Figure 4. Sedimentation assay SDS gels of human wildtype HARS and Y454S at 30**°C **and 40**°C. **(A)** SDS gels for hsHARS and Y454S at 30°C and 40°C as indicated. The first lane is the protein ladder (FroggaBio) with labelled molecular weights. Equal volumes of whole lysate (W), soluble fraction (S) and insoluble fraction (P) extracted from yeast variants in the sedimentation assay was loaded on to 15% SDS gel in 3 replicates. Gels imaged using the Gel Doc XR+ (Biorad). **(B) Western Blots of HARS and HARS Y454S.** Western Blot of sedimentation assay gels performed using primary anti-GFP rabbit monoclonal (ab32146) in 1:10000 dilution, and secondary antibody used was IRDye® 800CW Goat anti-Rabbit IgG (926-32211) in 1:10000 dilution. Blots were imaged using the Li- Cor Odyssey 9120 imaging system and quantified using ImageJ.

**Supplementary Figure 5. (A) Western blot of wildtype (hsHARS) and mutant HARS proteins.** Purified hsHARS, V133F, V155G, Y330C, S356N and Y454S HARS were separated by SDS-PAGE and transferred to PVDF membrane. Proteins were blotted using anti-6xHis primary antibodies First lane is the protein ladder (L) with labelled molecular weights. Expected mass of 6x-His tagged HARS is 58.23 kDa. **Melting curve of (B) V133F, (C) V155G, (D) Y330C, and (E) Y454S.** HARS protein were purified from *E. coli* using Ni^2+^ affinity chromatography. HARS protein samples were prepared with final concentration 1 μM in 96-plates for DSF. All samples performed in 4 replicates. Samples were heated from 25°C to 96°C at 1°C per minute and fluorescence intensity was measured. Standard error of each data point represented by lighter-colored error bars.  **T_M_ of (F) Y454S HARS and (G) hsHARS in apo form and with substrate supplementation.** Protein variants were incubated with substrates as indicated. Initial analysis was performed in the Protein Thermal Shift^TM^ software and data was fit to the Boltzmann equation to estimate the melting temperature (T_M_). Bars indicate the mean of 4 replicates ± the standard error**.** Significance levels are indicated using asterisks (****p<0.0001, **p<0.01, *p<0.05, ns = statistically non-significant).

**Supplementary Figure 6. Growth curve of Y454S mutant grown in (A) normal histidine 30°C, (B) normal histidine 40°C, (C) high histidine 40°C and (D) low histidine 40°C.** Yeast cultures were inoculated in a Synergy-H1 plate reader for 24 hours in 30°C with 10-min read intervals and 10 seconds shaking prior to each read. For each yeast strain there were 3 biological replicates and 3 technical replicates per biological. For His^++^, extra histidine (200mg/L) was supplemented in the growth media. For His^--^, histidine was reduced to 2mg/L from the normal growth media that contains 20mg/L histidine.

**Supplementary Figure 7. Sedimentation assay SDS gels of (A,B) HsHARS, (C,D)** **V133F, (E,F) V155G, (G,H) Y330C, and (I,J) S356N from cells grown under low (A,C,E,G,I) or high histidine (B,D,F,H,J) concentrations as indicated.** First lane is the protein ladder (FroggaBio) with labelled molecular weights. Equal volumes of whole lysate (W), soluble fraction (S) and insoluble fraction (P) extracted from different yeast variants in the sedimentation assay was loaded on to 15% SDS gel in 3 replicates. Gels imaged using the Gel Doc XR+ (Biorad).


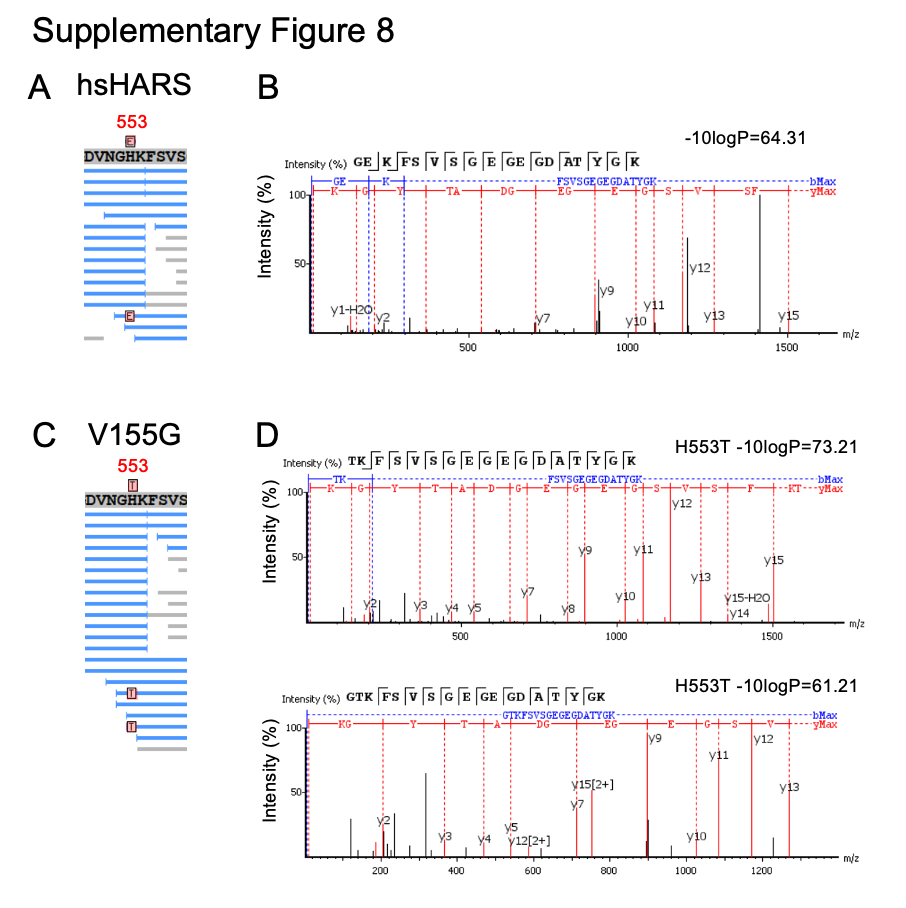


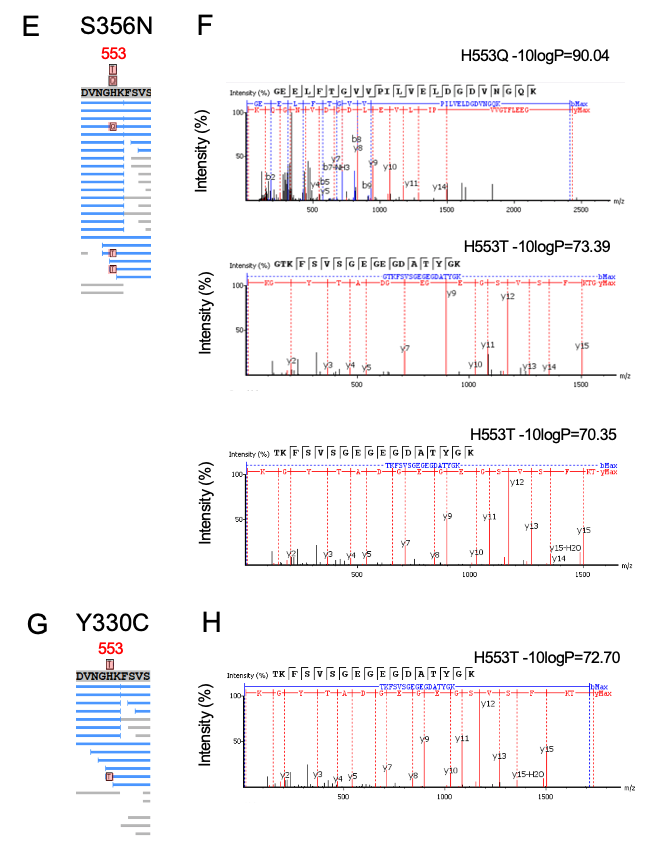


**Supplementary Figure 8**. **Mistranslation at HARS position 553.** Purified HARS-YFP expressed in hsHARS, V155G and S355N mutant yeast was in gel digested and submitted for mass spectrometry analysis at the Biological Mass Spectrometry Laboratory (The University of Western Ontario, London, Canada) to identify amino acids mis-incorporated in HARS-YFP. Protein coverage sequence of position 549 to 558 for **(A)** hsHARS, **(C)** V155G, **(E)** S355N and **(G)** Y330C yeast strains with mistranslation at H553T/Q highlighted. Corresponding peptide ion intensity spectra for **(B)** hsHARS, **(D)** V155G **(F)** S355N and **(H)** Y330C yeast strains with -10logP scores annotated.


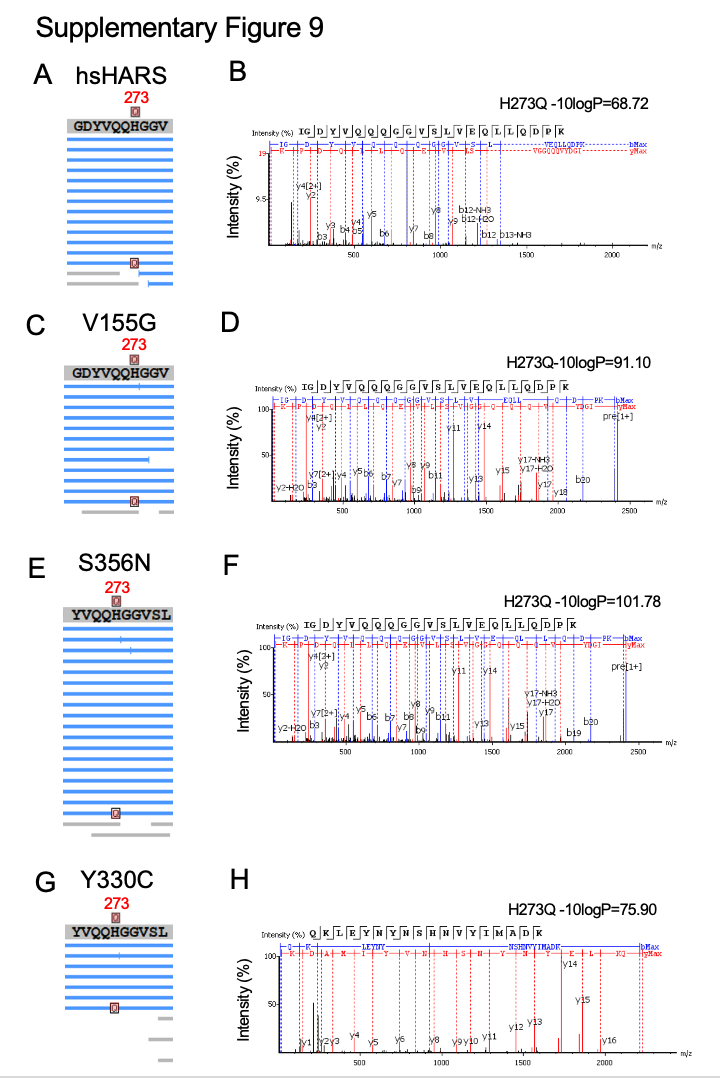


**Supplementary Figure 9.** **Mistranslation at HARS position 273.** Purified HARS-YFP expressed in hsHARS, V155G and S355N mutant yeast was in gel digested and submitted for mass spectrometry analysis at the Biological Mass Spectrometry Laboratory (The University of Western Ontario, London, Canada) to identify amino acids mis-incorporated in HARS-YFP. Protein coverage for **(A)** hsHARS, **(C)** V155G, **(E)** S355N and **(G)** Y330C yeast strains with mistranslation at H273Q highlighted. Corresponding peptide ion intensity spectra for **(B)** hsHARS, **(D)** V155G **(F)** S355N and **(H)** Y330C yeast strains with -10logP scores annotated.

**
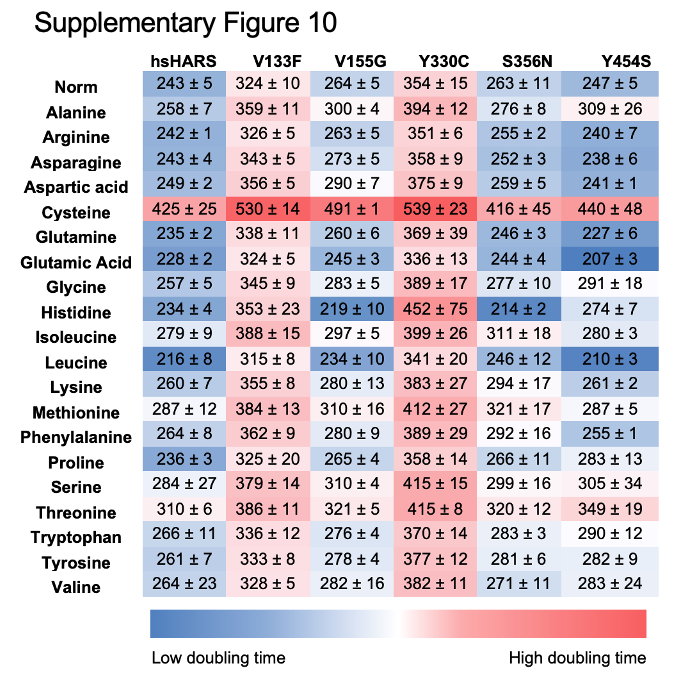
**

**Supplementary Figure 10. Doubling time of yeast supplemented with amino acids.** Yeast cultures were incubated in Synergy-H1 plate reader (BioTek) for 24 hours in 30°C with 10-min read intervals using 3 biological replicates and 3 technical replicates per biological. Fold change in doubling times comparing 200 mg/L amino acid-supplemented conditions to normal conditions for hsHARS and CMT-HARS. Blue represents low fold change in doubling time and red represents high fold change in doubling time.
